# Small flakes for sharp needs: Technological behaviour in the Lower Palaeolithic site of Marathousa 1, Greece

**DOI:** 10.1371/journal.pone.0324958

**Published:** 2025-06-30

**Authors:** Dalila De Caro, Moritz Kuhn, Nicholas Thompson, Eleni Panagopoulou, Katerina Harvati, Vangelis Tourloukis

**Affiliations:** 1 Paleoanthropology, Institute for Archaeological Sciences and Senckenberg Centre for Human Evolution and Paleoenvironment, Department of Geosciences, Eberhard Karls University of Tübingen, Tübingen, Germany; 2 UFG, Institute of Prehistory, Early History and Medieval Archaeology, Eberhard Karls University of Tübingen, Tübingen, Germany; 3 Hellenic Ministry of Culture and Sports, Ephorate of Paleoanthropology-Speleology, Athens, Greece; 4 DFG Centre for Advanced Studies “Words, Bones, Genes, Tools”, Eberhard Karls University of Tübingen, Tübingen, Germany; 5 Department of History and Archaeology, University of Ioannina, Greece; Universita degli Studi di Ferrara, ITALY

## Abstract

Marathousa 1 (~430 ka BP), located in the Megalopolis Basin, Greece, represents the earliest documented butchery site in the Southern Balkans, providing clear evidence of a direct association between artefacts and remains of *Palaeoloxodon antiquus*. The lithic assemblage features a distinctive small tools industry, primarily produced from local radiolarite, comprising both simple flakes and retouched tools. Through technological analysis, raw material characterisation, experimental knapping, and statistical analyses, this study explores how Middle Pleistocene hominins organised their technological behaviour as reflected in the lithic assemblage, and how these behaviours were shaped by the resource-rich setting of the Megalopolis Basin, characterised by abundant raw materials, water sources, and faunal availability. Results demonstrate the interplay between freehand and bipolar knapping, reflecting a flexible technological strategy to exploit the available radiolarite. Freehand percussion was mainly used in flake production, while the bipolar technique facilitated initial core reduction and late-stage exhaustion. The consistent microlithisation at the site is also evident in the exploitation of other locally available raw materials, such as limestone, flint, and quartz, supporting previous studies demonstrating small flakes’ effectiveness in diverse tasks. Technological patterns at Marathousa 1 broadly correspond to those observed at other Middle Pleistocene small tool sites; however, its distinctive intersection of raw material availability, technological choices and functional demands provides new insights into regional patterns of lithic variability across Eurasia during this period.

## Introduction

**Fig 1:**
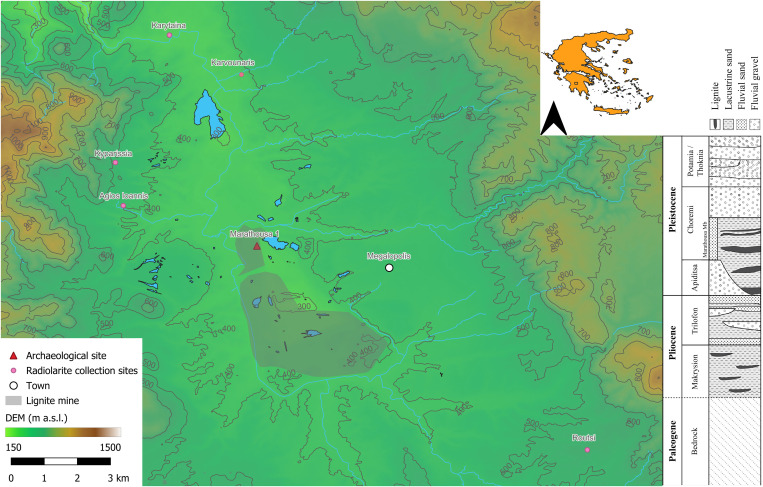
Location of Marathousa 1 and the localities where radiolarite for the experiment was collected in the Megalopolis basin (Peloponnese, Greece). The grey-shaded area marks the extent of the mines where lignite is being explored for commercial purposes. The elevation data are derived from the Shuttle Radar Topography Mission (SRTM) 1 Arc-Second Global DEM. Geographical features are based on OpenStreetMap (OSM) data using the OSM plugin in QGIS 3.34.10 [22]. The administrative boundary for the inset map of Greece is from the Natural Earth dataset (https://www.naturalearthdata.com/). The stratigraphic column is adapted from [23].

Marathousa 1, an open-air Lower Palaeolithic site located in the Megalopolis basin in central Peloponnese (Arcadia, Greece) (Fig 1), provides the opportunity to investigate hominin behaviour in southern Europe during the Middle Pleistocene. The site was discovered during a target-oriented survey conducted in 2012–2013, it dates to the Marine Isotope Stage (MIS) 12, ca. 430 ky ago [1–6], representing the oldest currently known archaeological site in Greece [2,7], though more recent discoveries of potentially older sites are being studied [8,9].

The Megalopolis basin is renowned for its rich fossil deposits [10,11]. The Pleistocene sequence is represented by the 200-meter-thick Choremi Formation, which includes two primary members: the Marathousa and Megalopolis Members (Mb). The Marathousa Mb is characterised by alternating layers of lacustrine clays, silts, and sands, interspersed with lignite seams, whereas the Megalopolis Mb is characterised by fluvial deposits [12]. The sedimentary sequence revealed important details about the paleoenvironmental conditions during the Middle Pleistocene. In particular, the presence of a paleolake played a vital role in sustaining the region’s ecosystem, ensuring freshwater availability during glacial periods, and acting as a *refugium* for large herbivores such as *Palaeoloxodon antiquus*, *Hippopotamus antiquus*, *Cervus elaphus*, *Bison* and *Dama* sp., as well as carnivores (*Canis*, *Vulpes*, *Felis*, *Mustela* sp.), and primates (*Macaca*) [6–17]. The associated flora, including trees like *Acer*, *Alnus*, and *Salix*, as well as aquatic and flowering plants like *Mentha aquatica*, *Verbena officinalis*, *Nymphaea alba* reflects a temperate landscape that likely provided a rich array of resources for Middle Pleistocene hominins [15]. The region’s river systems also provided aquatic resources and naturally transported knappable materials, readily available along the basin’s streams, such as radiolarite, quartz, flint and limestone pebbles, exploited for tool production. Another key feature of Marathousa 1 is the stratigraphic and spatial association of lithic artefacts with faunal remains, particularly *Palaeoloxodon antiquus*, bearing cut marks and percussion damage [2,13], indicative of butchery and other subsistence-related activities, underscoring its significance as a resource. While the absence of hominin fossils (although an individual likely belonging to the Neanderthal lineage has been recovered from Middle Pleistocene deposits in the Megalopolis basin [18]) precludes direct insights into the paleoanthropological profile of the individuals who inhabited the area, the lithic assemblage offers a relevant proxy for exploring technological and ecological strategies during this critical phase of human evolution [19]. A “small tool” component characterises the lithic industry in Marathousa 1, a reduction modality that implies the intentional production of retouched and unretouched artefacts, usually smaller than 40 mm [20]. This technological pattern aligns with several Middle Pleistocene sites where small tool production is often associated with aquatic environments and megafauna exploitation, particularly of elephants. However, the specific technological and contextual attributes of Marathousa 1 within this broader setting remain to be fully explored. This study investigates the specific characteristics that define the lithic assemblage from Marathousa 1 in relation to the broader technological and ecological context outlined above. Combining typo-technological analyses, experimental knapping, and raw material units (RMU) analyses, we reconstruct the *chaînes opératoires* followed at the site. Evidence of freehand and bipolar knapping is examined to assess the role of each of the two techniques within the technological system at Marathousa 1, considering at the same time both the properties of the local radiolarite and the choices shaping lithic production. Our study addresses the relative contribution of freehand and bipolar techniques to evaluate the technological choices and causal parameters engaged in the production of small-sized blanks. Specifically, we investigate the roles of raw material properties and pebble size and assess to what extent the small size of blanks is a result of bipolar production. While these questions have been discussed by other researchers working on ‘small-tool assemblages’, they have rarely been tested through experimental approaches –hence the significance of the experimental component presented here. More broadly, by analysing the compositional variability of this important site, this study aspires to refine our understanding of technological flexibility among Middle Pleistocene hominins in the southern Balkans, a region that served as a crossroads for hominin population movements to and from Europe [21].

## Materials and methods

### The lithic assemblage of Marathousa 1

Systematic excavations at Marathousa 1, spanning seven field seasons from 2013 to 2019, targeted a total area of 72 m² rich in lithics that were found in spatial and stratigraphic association with faunal remains. Evaluated both macroscopically and microscopically, the artefacts are, with few exceptions, remarkably well-preserved, bearing very limited (if at all, of low intensity) mechanical surface alterations such as edge damage marks [24,25]. This evidence matches well the assessment of the depositional dynamics and site formation processes, as deduced from the sedimentological and lithostratigraphic analysis [26], the study of the site’s spatial taphonomy [27], the taphonomic evidence on the faunal remains (*e.g.,* bones in anatomical association) [13,14], and the anisotropy of magnetic susceptibility [5]: artefacts and osseous material were entrained by a mudflow or hyper-concentrated flow that slightly re-distributed them *en masse* and only locally; overall, the archaeological record was not exposed to the subaerial elements for a considerable amount of time. The lithic assemblage is stored in Athens at the Ephoreia of Palaeoanthropology-Speleology (Ministry of Culture and Sports, Greece). The artefacts (N = 2327) are predominantly produced using the locally abundant radiolarite and can be classified into the following technological classes: flakes (≧ 15 mm: 14%, N = 318), tools (3%, N = 70), cores (1%, N = 29), chips (10–14 mm: 66%, N = 1531), microchips (< 10 mm: 5%, N = 113), and debris (11%, N = 266). Chips and microchips refer to small knapping waste that exhibit clear features of controlled flake detachment, such as identifiable platforms or ventral surfaces. In contrast, debris consists of irregular lithic fragments lacking diagnostic technological attributes, often resulting from accidental breakage, raw material flaws, or core shattering during percussion [28,29]. A detailed explanation of the essential terminology used in this study can be found in the S1 Appendix. Artefact dimensions range from an average of 20 mm for unretouched flakes, to 26 mm for retouched tools, thereby indicating a flake-based “small tool” industry [20,24]. The trend towards small debitage is underscored by a high presence of chips and micro-chips, comprising 71% of the total. Among these, only the longest specimens, measuring 10–15 mm and with an acute cutting edge angle of at least 60°, are considered to meet “*... the absolute limit of credible tool size*” ( [30], p. 17), and were thus thought to have had potential functionality, representing “target products” (see also [24] for a relevant discussion).

In describing the technological traits of the lithic assemblage, we considered all the complete and almost complete flakes (N = 159), tools (N = 72), cores (N = 29), and a sample of chips ≧ 10 mm (N = 68), for a total of 328 artefacts. For the multivariate statistical analyses (see below), we focused only on fully complete and unretouched radiolarite flakes ≧ 15 mm (N = 90). Finally, in assessing the exploitation of raw materials through RMU identification, we also included the fragmented flakes and the other technological classes (chips, microchips, and debris).

### Technological analysis

**Fig 2:**
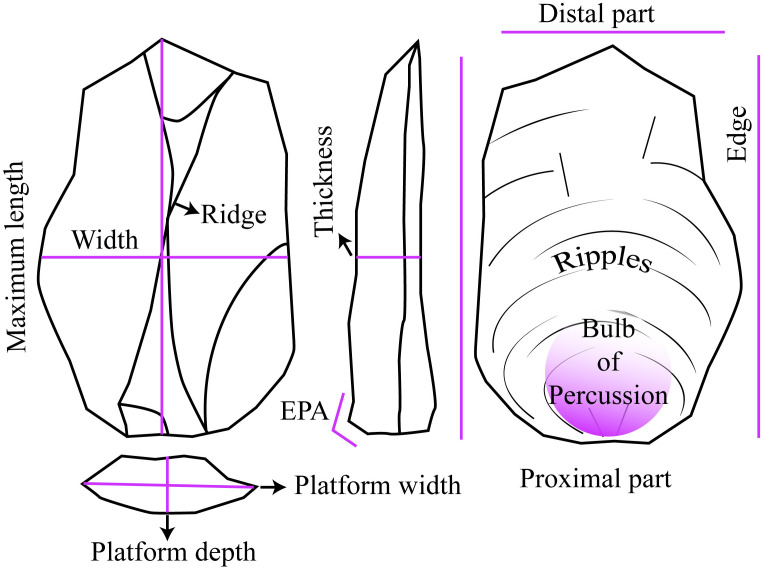
Attributes considered for technological analyses.

All lithic artefacts in this study, including both the archaeological assemblage and the experimentally produced flakes, were analysed using qualitative and quantitative technological assessments [28,31,32]. For archaeological flakes, tools, and cores, we recorded more detailed qualitative and quantitative data, including the level of completeness, mass (g), platform type, exterior platform angle (EPA), bulb of percussion (absent, diffuse, pronounced, hinge) and flake termination (feather, hinge, step). For the linear measurements on complete flakes, we considered the maximum length (from proximal to distal part), the width (perpendicular to the length at the midpoint), and thickness (perpendicular to the point of conjunction between length and width, at the median part of the flake) [33]. We also measured the platform depth at the midpoint, and the line extending from one edge to the other determined the platform width (Fig 2). Moreover, we noted any occurrence of knapping accidents and specified the type of accident (*e.g.,* Siret fracture). We also recorded the cortex, the dorsal scar patterns, and the number of cutting edges. The latter were recorded if they were equal to or longer than 10 mm and with an angle equal to or less than 50° [32,34]. We counted them considering a hypothetical flake with a maximum of three sharp edges, excluding the platform. We also recorded the presence of the central convexity (natural, mid-dorsal ridge or crest-like), the presence/absence of platform trimming, and the type of ripples. To identify bipolar pieces in the archaeological assemblage, we searched for a combination of attributes such as crushing and fissuring on opposed ends (especially on cores), unparallel ripples, flat ventral surfaces, Siret fractures, and step terminations. For example, flakes exhibiting a pronounced bulb of percussion, platform preparation, and regular ripple patterns were likely produced through freehand knapping. In contrast, artefacts with flat ventral surfaces, step or hinge terminations, bidirectional flaking pattern, broken and linear butts, lack of bulb, and deep and irregular ripples could have been produced through bipolar percussion.

In the statistical dataset, which includes only radiolarite flakes, these attributes were recorded individually according to the scheme presented in Table 1, with each flake assigned to either bipolar or freehand categories based on the known knapping technique used in the experiment. In contrast, archaeological flakes were recorded solely based on their attributes, without pre-assigning them to a reduction technique group (freehand or bipolar).

**Table 1:** Attribute Definitions and Scoring Criteria for Flake Analysis.

Variable	Type	Description	Scores
Type of flake termination	Nominal	Different types of terminations observed on the flakes.	0 = Other, 1 = Feather, 2 = Plunging, 3 = Hinge, 4 = Step
Bulb of percussion	Nominal	Type of bulb on the ventral surface.	0 = Hinge, 1 = Flat, 2 = Moderate, 3 = Pronounced
Platform preparation and trimming	Nominal	Whether the platform was prepared/trimmed before knapping.	0 = Absent, 1 = Present
Central convexity of the flake	Nominal	The type of central convexity on the dorsal surface.	0 = Absence of convexity, 1 = Central and subcentral ridge, 2 = Natural convexity, 3 = Crest and semi-crest
Number of sharp edges	Ordinal	Count of sharp edges on the flake, ranked in order.	0 to 3, considering the distal
Type of ripples	Nominal	Different types of ripples observed on the ventral surface.	0 = Absent, 1 = Closely spaced. More directions. 2 = Parallel, widely spaced

Retouched tools were analysed from a technological perspective, while the terminology used for their classification follows [35]. Finally, to support the technological analysis and to infer the reduction sequences, we aimed to identify refits and conjoins in the archaeological assemblage, along with the “*Lecture technologique*” and the “*Remontage mental*”. The latter refers to the process of visualising and reconstructing the original positions of flakes within the core or among each other ( [36,37]; see also [38] for an application of this approach).

In the context of the experimental program, and in order for us to further explore the importance and implications of each reduction sequence, we defined an additional parameter, defined here as “Persistence”. This represents the number of unsuccessful attempts (trials) a knapper performs to detach a piece (flake or chip) that is at least longer than 10 mm, thus leaving easily identifiable traces on cores and blanks. This parameter was used to explore and discuss the advantages of choosing one percussion technique over the other. Nevertheless, these mechanical imprints on the artefact’s surfaces could be mistaken, within the archaeological context, for those resulting from battering, a characteristic often linked with bipolar percussion. Due to this potential overlap, we did not include this parameter for the archaeological assemblage to hypothesise the knapping techniques, as it is difficult to determine whether these types of traces found on a few artefacts were due to the knapper´s persistence stemming from a lack of expertise, post-depositional processes, or the battering resulting from the employed flaking technique.

We refitted 13 experimental cores to better understand the knapping dynamics of each reduction strategy. We focused on cores and flakes sharing technological characteristics or morphological attributes with those from Marathousa 1 (*e.g.,* flakes with a thicker edge opposite a sharper one, often with centripetal scars, irregular flat and/or elongated shapes, sub-rounded segments, and fully exhausted centripetal cores). This process was also useful for understanding the difficulty of achieving these goals for the specific raw material used (radiolarite pebbles from the Megalopolis basin, see below). Flint refits are common, whereas quartz refits present analytical challenges due to the lack of conchoidal fracture, unpredictability, and quick dulling of the tools [39,40]. In contrast, the radiolarite found in the Megalopolis basin could potentially pose an intermediate level of challenge. This is due to the simultaneous presence of conchoidal fracture, which yields regular pieces, and factors such as calcite and quartz veins, impurities, and intersecting cleavage planes, which result in irregular, often chunky blanks. These blanks frequently exhibit a relatively high degree of fragmentation and shatter-like morphologies (all details regarding the analyses on experimental products are in S2 Appendix).

### Experimental analysis

One of the objectives of this study is to assess the relative preference of the freehand *versus* the bipolar technique in Marathousa 1, addressing how the two different techniques may have been employed as technological responses to various knapping decisions. Freehand knapping is defined as the direct percussion of a core secured in one hand, using a hammerstone manipulated by the other hand. The formation of flakes, mental schemes and mechanical assumptions related to this technique have been broadly investigated during the last decades (*e.g.,* [28,41,42]). The bipolar-on-anvil (or simply “bipolar”) technique was first recognised in the early 20th century and defined as the production of flakes by resting a core on the anvil and striking it with a hammerstone [31,43–45]. This knapping strategy was rather ubiquitous during prehistory, likely due to its relative immediacy in the actions to be applied and its easy transmission in knapping learning environments [30,46,47]. On this basis, the bipolar technique “*... should be seen as simple, but not simplistic*”, considering that its simplicity does not necessarily imply crudeness ( [30], pp. 63−62). It is particularly efficient for producing small tools from small cores and opening rounded pebbles, which lack a striking platform with convenient angles [48,49].

The analysis of the archaeological assemblage may indicate the possible presence of bipolar knapping at Marathousa 1, according to attributes (see above) and established technological criteria (*e.g.,* [28,41,47,48,50–62]). However, as not all diagnostic traces of bipolar knapping consistently appear on artefacts [31,53,63], its presence in archaeological contexts may be underestimated. To refine our understanding of its role in this context, we conducted an experiment to identify tendencies, variability, and subtle differences between bipolar and freehand flakes. Multivariate analysis was applied, as this method allows for a more comprehensive examination of technological trends beyond a simple presence/absence classification. This approach aimed to provide a more detailed assessment of the relationship between bipolar and freehand knapping at Marathousa 1 regarding flake production. Radiolarite was by far the most preferred raw material (88% of all pieces) at Marathousa, leading us to focus on this rock type. The secondary deposits of the Megalopolis Basin yield abundant radiolarite pebbles (cf. [3,4,24]), with qualitative and mechanical properties closely matching those of the archaeological assemblage. To ensure the accuracy and relevance of our experimental results, we used radiolarite and hammerstones collected from these deposits (see Tables A and B in S2 Appendix). The raw materials utilised in the experiment were collected from different locations in the Megalopolis Basin (Fig 1).

### Experimental design

The experiment addressed two main questions:

1. Are there quantitative and/or qualitative traits of flakes which are diagnostic to accurately distinguish the reduction strategy (freehand or bipolar)?
2. Which reduction strategy was primarily used to produce flakes at Marathousa 1?

We collected 91 radiolarite pebbles from river deposits in the northern and western part of the Megalopolis basin (Karytaina, Kyparissia, Karvounari, Agios Ioannis, and Routsi), including one sample from a primary outcrop, to test its knappability. Each pebble’s dimensions, volume and mass were recorded and photographed before knapping. The collected pebbles had an average length of 68 mm, width of 48 mm, and thickness of 34 mm, with an average volume of 138 mm³ and an average mass of 280 g (Table A S2 Appendix). To maintain consistency, we selected pebbles with a homogeneous surface texture for the experiments, except for the sample from the primary outcrop. The participants included one expert knapper and four moderately experienced individuals with extensive theoretical knowledge of percussion techniques and Palaeolithic chrono-cultures. We chose a slightly heterogeneous level of practical expertise to reflect the likely variability in skill levels among hominin groups. Instead of assessing individual expertise, our goal was to identify which knapping technique most plausibly produced flakes comparable in size and morphotypes to those in the archaeological assemblage. Volunteers extensively examined photographs of the Marathousa 1 lithic assemblage to understand the target flake morphologies. They then received general guidelines on replicating those forms through different reduction strategies based on knapping techniques, with the aim of approximating the archaeological variability, ensuring consistency while allowing participants some flexibility in selecting pieces for percussion. For freehand percussion, they minimally prepared the core and applied different reduction sequences according to knapping platforms and flaking surfaces, recording each step until the core was exploited as far as its properties allowed. For bipolar percussion, they struck the core while it rested on the anvil, noting any changes in striking platforms or flaking surfaces until further reduction was no longer feasible. Because radiolarite cores at Marathousa 1 are intensively reduced, with maximum lengths ranging from 18 mm to 37 mm, our experimental goal was to replicate that pattern by applying the reduction modes identified in the archaeological assemblage: unidirectional, bidirectional, multidirectional, and centripetal. Cores were considered *exhausted* when further knapping could only produce chips or microchips and *fully reduced* when the final strike caused the core to fragment into flakes and debris (Fig B S2 Appendix). Additionally, cores were abandoned when internal flaws, such as fractures, impurities, or structural defects, prevented effective flake detachment.

Each percussion movement was counted and recorded separately, performed using one of the eight pre-selected hammerstones. For each session, the participants could choose among various hammerstones and work down the pebbles with reduction sequences combined in the following five ways:

1. Bipolar
2. Freehand
3. Bipolar-Freehand
4. Freehand-Bipolar-Freehand
5. Bipolar-Freehand-Bipolar

For the application of the bipolar-on-anvil knapping, we followed Hiscock’s [48] strict definition, which requires the core to be immobilised and compressed between the hammerstone and the anvil before the strike. To maintain compression forces, cores were always positioned to ensure stable contact with the anvil, allowing for bidirectional compression (Fig C S2 Appendix) (refer to [53] for relevant discussions on bipolar fracture types). This approach differs from non-axial methods [53,64–66], which, despite being appropriate for knapping small pieces, exhibit fracture mechanics similar to freehand [48,62], potentially biasing comparative analyses [67]. To assess the potential presence of hybrid bipolar approaches at Marathousa 1, we asked participants to also include anvil-assisted bipolar (B.A.A.) percussion in the experiment. This established a control group for our comparative analyses, allowing for the identification of any differences between B.A.A. and the two main techniques (bipolar-on-anvil “axial”, and freehand).

**Fig 3:**
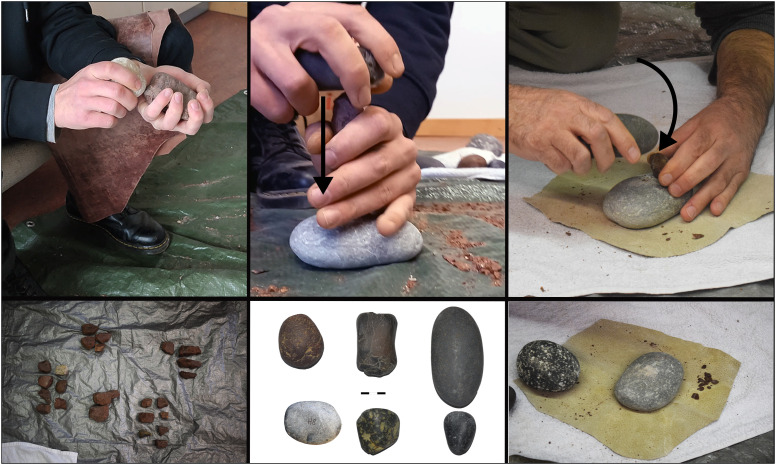
Experiment overview. These pictures depict examples of the tasks by the authors. The first row represents the three percussion techniques utilised: freehand, bipolar-on-anvil (*sensu* [48]), and bipolar anvil-assisted. The second row shows a sample of the pebbles collected in the Megalopolis basin, the hammerstones and the anvil utilised for bipolar techniques.

Throughout the experiment, the use of plastic nylon enabled the collection of all chips, chunks, and flakes from each core. Each percussion episode, with its associated products and cores, was placed into a separate bag, labelled by the number assigned to each cobble/pebble (1, 5, 90, etc.). Within each bag, we separated the groups of products according to the different techniques adopted by the participants during core exploitation, numbering them respectively (*e.g.,* RS1, 2, 3, etc). Additionally, we utilised smaller bags to separate the flakes produced from each successful strike, further numbered progressively. Among all the pebbles collected for the experiment in the Megalopolis basin, 51 were utilised by the volunteers during the four-day experiment (Table C S2 Appendix). The remaining pieces were tested and discarded because of their general unsuitability for knapping. Fig 3 and Fig C S2 Appendix present examples of the gestures and reduction sequences.

### Participant recruitment and consent

The experiment (including recruitment and controlled percussion sessions) occurred between January and April 2023, in the framework of stone tool-using experiments approved by the University of Tübingen’s Ethics Commission. Written informed consent was obtained from all participants before the study. In line with the recommendations of our Ethics Commission, all safety measures (including the use of protective gloves and goggles) were followed during the percussion movements. The experimental process was in line with the principles of the Declaration of Helsinki.

### Statistical analyses

IBM SPSS Statistics v. 29 [68] was used for multivariate analyses to characterise freehand and bipolar flakes in the experiment and to plot the archaeological radiolarite flakes, allowing for the assessment of reduction strategies. Following Vergès & Ollé [53], who argue that “bipolar knapping does not exist” (2011: 1017) in the sense of being a singular, static category, but rather encompasses a range of patterns and outcomes, we adopted a multivariate approach aimed at identifying combinations of traits that best characterise the knapping techniques targeted in this study. Initially, we excluded one extreme outlier in the archaeological assemblage (a flake of 45 mm length, see Fig A in S1 Appendix), detected using boxplots and the interquartile range approach in Past (v.4.10) [69]. The first analysis was a Principal Component Analysis (PCA) on continuous, quantitative variables (length, width, thickness of the flakes, width and depth of the platform) using the correlation matrix due to the substantially varying ranges across variables (for example, with lengths generally much greater than thicknesses or platform depths). The correlation matrix standardises the data, ensuring that each variable contributes equally to the analysis, regardless of its original range. The second analysis was a Categorical Principal Component Analysis (CATPCA) on qualitative traits. This is a non-linear approach relying on the optimal scaling procedure [70,71], which allows for the multivariate analysis of qualitative variables. The optimal scaling procedure in SPSS quantifies these categorical variables by assigning numerical values that maximise the variance explained by the principal components [70,71]. In this way, CATPCA can be used on lithic mechanical and functional variables (as previously defined by [58]). In this study, the nominal and ordinal categorical variables included were the type of flake termination, bulb of percussion, presence of platform preparation and trimming, central convexity of the flake, number of sharp edges, and type of ripples (see Table 1), following the framework established by Cotterell and Kamminga (1987) and de la Peña (2015) [41,50]. In the plots of the two multivariate analyses, a different colour and shape were used to denote flakes (cases) associated with the bipolar knapping technique (dark blue rings), the freehand technique (pink circles), and the archaeological samples (green rhombi). The number of PCs or CATPCs to plot in each case was decided based on the scree-plot “elbow” criterion (*e.g.,* [70]). The observed variation among the three groups on each PC axis was interpreted based on the factor loadings of the original variables [70,71].

Finally, to statistically evaluate our observations of clear differences across the groups (representing the percussion techniques and the archaeological assemblage) in the PCA plots, we additionally compared their scores on each plotted PC axis using four Kruskal Wallis tests (corresponding to the PC1 and PC2 scores of each of the multivariate analyses) [70]. For the tests showing a statistically significant omnibus p-value (P < 0.05), pairwise Mann-Whitney U tests were used to identify the exact group differences within the dataset. These non-parametric tests were preferred because some PCs were not normally distributed and/or contained outliers.

### Raw material unit analyses

When analysing the archaeological assemblage, we aimed to recognise Raw Material Units (RMUs) [72–74] representing individual pebbles or cobbles exploited at the site. Cores and debitage identified as belonging to the same pebble or cobble comprise an RMU. We classify these units based on technological traits and raw material attributes. In particular, we analysed the macroscopic characteristics of the artefacts that compose an RMU to infer the technological stage in which specimens were imported at the site, as well as to assess identified and/or anticipated on-site and off-site activities, based on missed pieces in RMU [75]. The artefacts were sorted by types of radiolarite, the latter categorised according to their features, texture, colour patterns, and shades as per the Munsell tables. If present, we also recorded the types of inclusions and the appearance and texture of the natural surfaces and cortex. Additionally, we documented the presence and types of secondary colours/inclusions, such as black bands (whether thick, thin, parallel, or random) and mottling. The identification process was based on the texture of the radiolarite, noting that areas closer to the cortex or to the pebble’s external parts might exhibit a more coarse-grained texture. In contrast, the inner parts are usually more homogeneous. We recognised pieces bearing intermediate granularity, which aided in the process of refitting, together with the abovementioned elements. We also considered techniques applied, reduction strategies followed, or specific technological patterns like platform preparation. These were integrated with all the previously mentioned macroscopic elements. As a result, we formed a set number of clusters, and each cluster was assigned a unique numeric code corresponding to a specific combination of macroscopic and technological characteristics. These clusters, comprising a minimum number of two specimens, facilitated the recognition of the RMUs and, in some instances, promoted refitting [75–78]. The RMU analysis, combined with technological, experimental, and statistical results, enabled the reconstruction of *chaînes opératoires*, contextualising technological choices and lithic production dynamics at Marathousa 1.

## Results

### The archaeological assemblage

**Fig 4:**
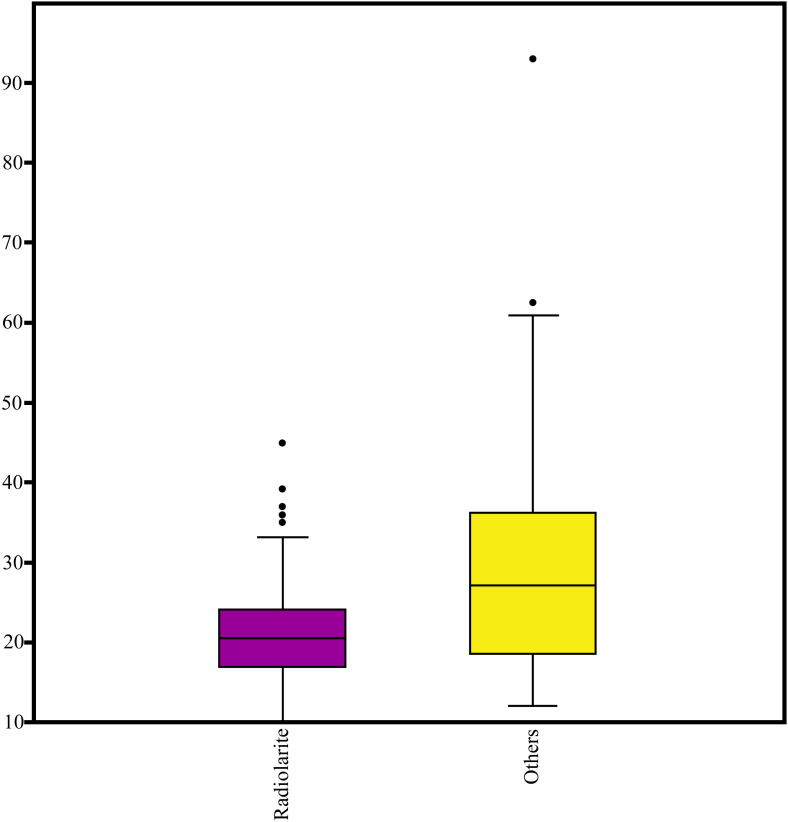
Boxplot showing the differences in length (mm), when comparing artefacts made on radiolarite to those made on other raw materials.

The analysed archaeological assemblage included flakes, retouched tools, cores, and a sample of the chips. The average dimensions of complete flakes, regardless of the raw material, are 21.08 mm x 17.62 mm x 6.89 mm, with an average mass of 5.15 g. Considering only the complete radiolarite flakes, the mean dimensions are 19.72 mm x 16.75 mm x 6.65 mm, and the mass is 3.49 g (Fig 4; Figs C and D in S1 Appendix). The tools, which have average dimensions of 22.37 mm x 16.98 mm x 8.74 mm and a mass of 4.82 g, are manufactured on flakes or chips (N = 41), debris, or exhausted cores (N = 30).

**Fig 5:**
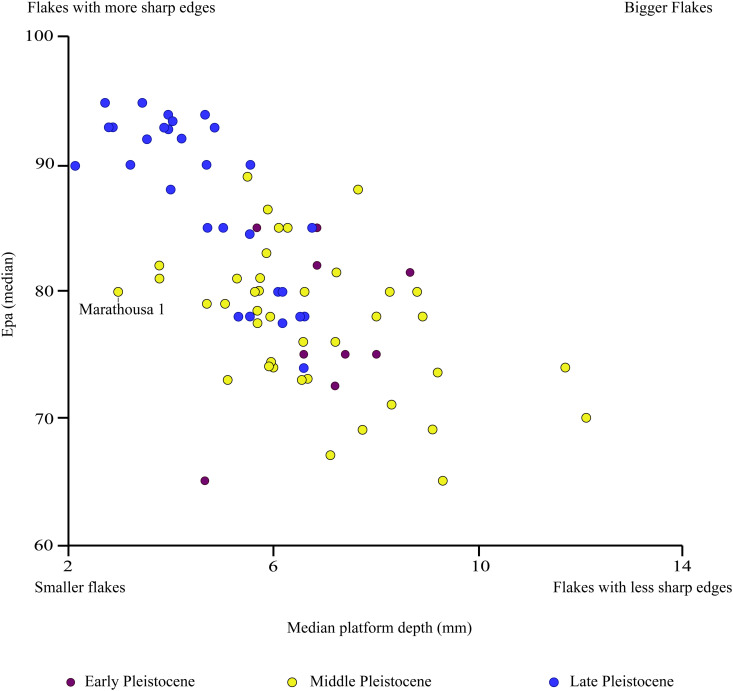
Scatter plot comparing the median values of exterior platform angle (EPA) and platform width (based on data published in [76]; see link to open access data below). Bigger flakes in the plot indicate greater platform depth and broader external angle. From this perspective, Marathousa 1 shows very small flake dimensions compared to other Middle Pleistocene sites, while its median platform depth mostly overlaps with Late Pleistocene sites [76] (data available at https://zenodo.org/record/1408081#.W6iyn84zaHs).

Among flakes, chips and tools, the most common platform type is plain (N = 83), followed by linear (N = 25), faceted (N = 18), punctiform (N = 4), and other types such as natural, battered, dihedral and on cleavage (N = 32). The median platform depth is 3 mm, and the median EPA is 80°. These two values place the flake production of Marathousa 1 among the smaller flakes with a significant number of sharp edges, according to the schema proposed by Ž. Režek and colleagues ([76], Fig 5). For flakes and chips (≧ 10 mm), the ratio of sharp edges to the total number of retouched and unretouched artefacts is 1.79, with a standard deviation (σ) of 0.80. However, when we only consider the unretouched artefacts, this ratio increases to 1.96 (σ 0.75).

**Fig 6:**
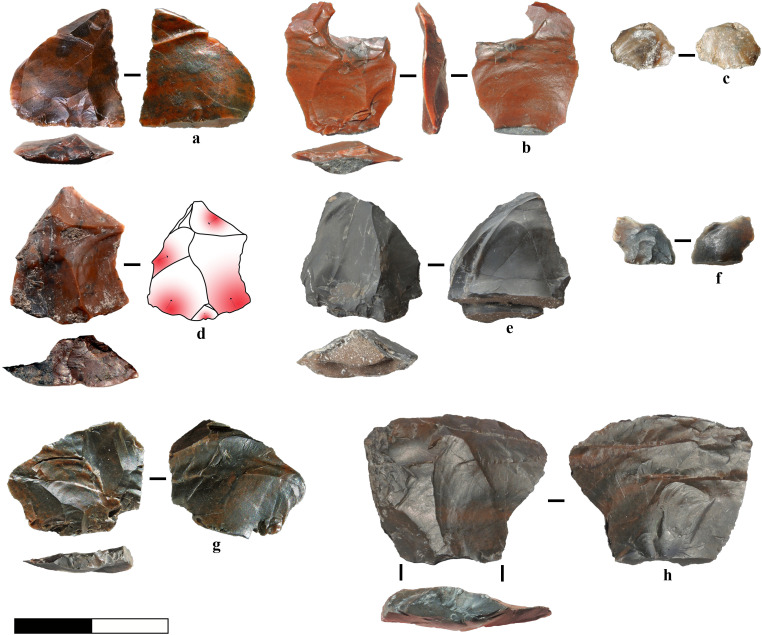
Target flakes in radiolarite. a) Flake with platform preparation and traces of proximal trimming, possibly detached to assess the flaking surface after a step fracture visible on the negative of a previous unidirectional removal. b) Target flake or edge modification flake with proximal preparation, unidirectional pattern and natural notch on the distal due to an impurity. c) Chip from retouch. d) Flake with platform preparation and central convexity produced through a centripetal pattern. e) Flake with sub-central ridge and double platform due to the presence of cleavage planes. f) Chip from retouch. g) Target or retouch flake with platform preparation. 8) Production or confection flake with orthogonal negative on the dorsal surface.

**Fig 7:**
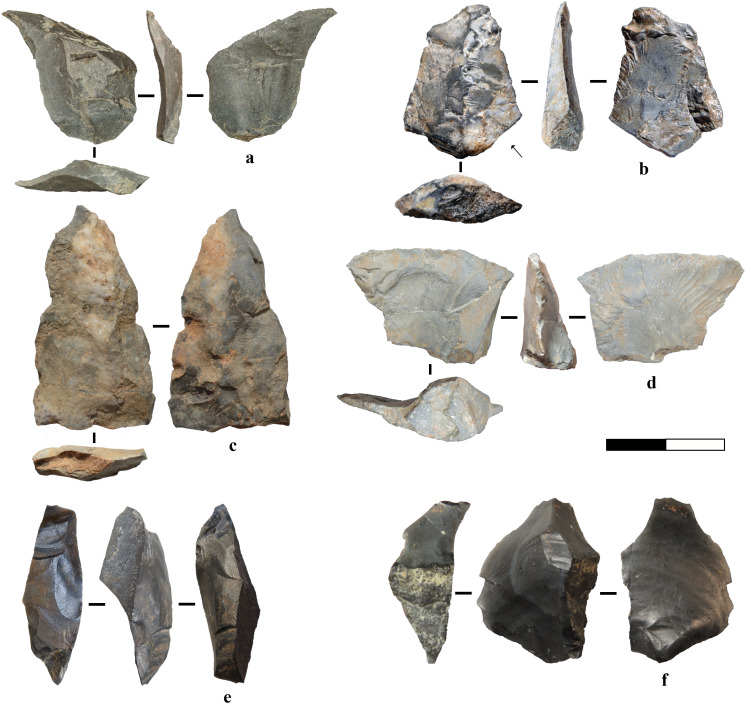
Flakes in other raw materials. a) Mudstone flake. b) Limestone point from centripetal reduction. c) Limestone elongated pointed flake with unidirectional pattern and use-wear traces. d) Limestone robust flake. e-f) Flint flakes obtained from bipolar percussion.

In flakes and chips, dorsal scar patterns primarily exhibit unidirectional and unidirectional convergent orientations (55%), followed by multidirectional (12%), bidirectional or opposite (12%), orthogonal (8%), centripetal (4%) and other variations (1%). Approximately 33% of the assemblage exhibit cortex and natural surfaces, from subangular pebbles. Many flakes and tools are characterised by a central ridge or crest, facilitating the direction of the force of propagation from the impact point to the distal part, thereby favouring, on some occasions, the production of sharp edges. Among the vast majority of flakes obtained under unstandardised strategies, we highlight the presence of small radiolarite flakes with platform preparation and centripetal scar patterns that permit the formation of central convexity and, in some cases, convergent edges (Fig 6), as well as the production of unretouched limestone points, produced through the previous removals of two parallel flakes or producing target flakes, possibly from centripetal knapping (Fig 7b, c).

**Fig 8:**
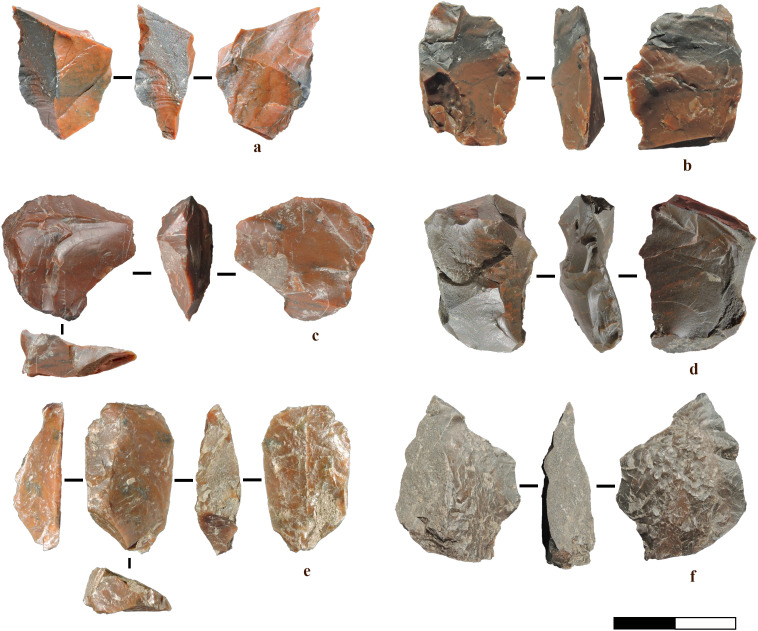
Naturally backed knives and core edge elements (1-6).

Alternatively, this central or sub-central ridge may result from the intersection of the flaking surface with the side of the core, often leading to a thicker edge opposite to a sharp one. Such naturally backed knives (N = 22), along with those obtained by retouch (N = 9), appear to be a primary objective of knapping at Marathousa 1, together with the production of simple flakes, which are usually left unretouched. Unretouched backed knives are obtained from core edges, utilising cleavage planes or knapping accidents like Siret fractures (Fig 8). Conversely, backed knives produced with steep retouch opposite to a sharp, unretouched edge are often made on tabular radiolarite blanks, which are reshaped to achieve desired forms. Similar processes apply to some scrapers, a type of tool relatively abundant in the archaeological assemblage, manufactured from heavily retouched debris and flakes, often in combination with composite tools like borers (N = 24). A further tool category comprises notched and denticulated pieces (N = 22) produced on both flake blanks and debris. Additionally, other tool types include flakes retouched on the ventral proximal surface (thinning) and burin-like artefacts.

**Fig 9:**
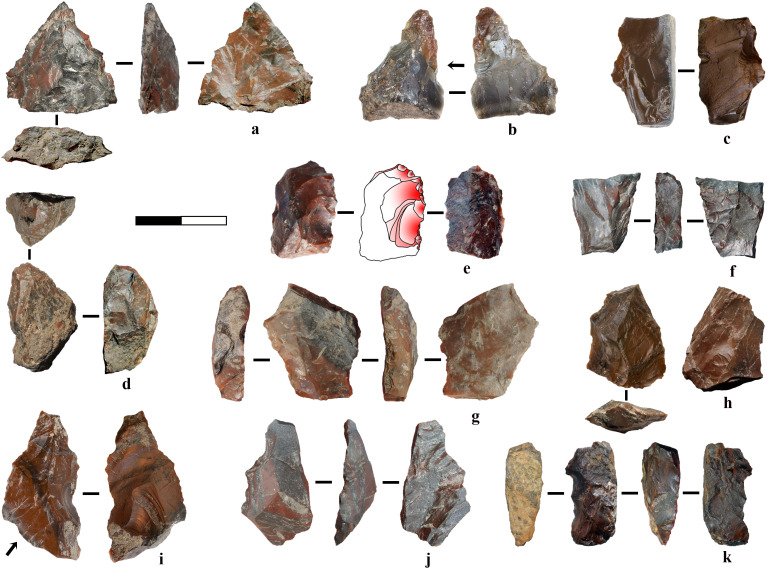
Retouched tools. a) Convergent denticulate on flake. b) Median flake fragment retouched in convergent tool with resharpening traces. The arrow indicates the axis of percussion. c) Retouched tool on a cortical piece, with a natural back opposite a notched edge. d) Scraper on a semicortical pebble fragment. Flake and chip negatives exhibit patterns consistent with those in the assemblage. e) Carinated scraper on a subangular pebble fragment. The negatives of flakes and chips produced during retouching exhibit patterns consistent with those observed in the assemblage. f) Retouched tool with a rectilinear edge opposite a backed edge. g) Retouched pointed tool. h-i) Denticulate on flake. j) Notch on cortical bipolar flake. k) Heavily reduced denticulate on a cortical pebble fragment.

**Fig 10:**
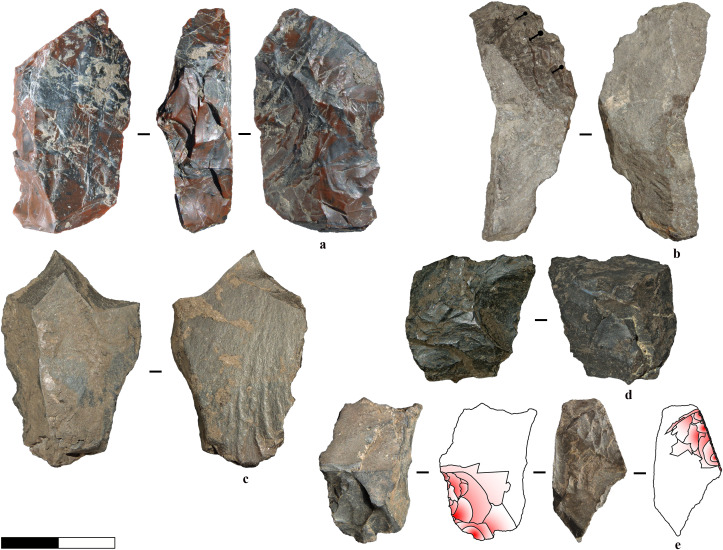
Expedient tools on debris and natural blanks. a) Backed knife on radiolarite tabular piece. b) Notch on limestone blank. c) Pointed tool obtained through two alternate removals on tabular mudstone blank. d) Notch on chunk with convergent shape. e) Chunk with serrated retouch (and possibly use-wear) on two larger alternate removals which form a spine. The removals occur on two opposite surfaces (from the cleavage plane and the side of the piece).

The retouched tools are generally characterised by larger dimensions than the flakes, primarily due to two main factors. First, they are produced during the initial phases of reduction, wherein the core volume is larger than during subsequent production phases (see Fig 9c, f, j, k). Consequently, these tools often retain cortex or natural surfaces, accounting for approximately 54% of the samples. Second, another more expedient method for swiftly manufacturing tools to address immediate tasks involves shaping by-products and natural blanks, sometimes with an already suitable shape conducive to prehensility (Figs 9 and 10).

**Fig 11:**
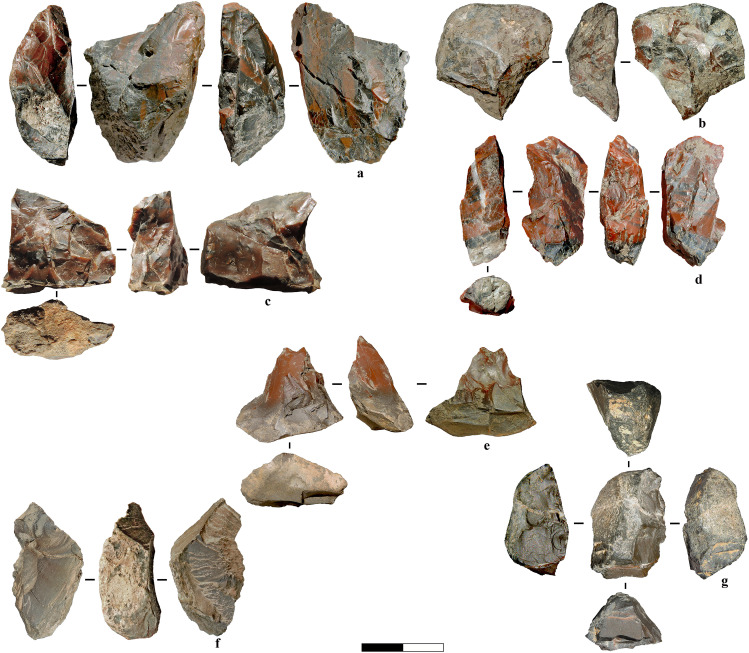
Cortical flakes and debris. The pieces generally originate from pebble fragments during the initial stages of reduction, except for d, which possibly represents a cortical exhausted bipolar core oriented vertically.

Although we treated the archaeological assemblage as a whole when analysing general technological patterns, we also recorded the likely knapping technique (freehand or bipolar) associated with individual artefacts, when possible. These internal annotations served as qualitative indicators and were not considered in the statistical testing. Among the 328 artefacts included in the technological analysis, we identified 223 pieces (68%) as tentatively attributable to freehand percussion and 63 pieces (19%) to bipolar percussion. The remainder were left undetermined. Cortex is present on 91 freehand-attributed artefacts (41%) and 24 bipolar-attributed artefacts (38%), with the latter often associated with flakes and debris showing sub-rounded pebble surfaces (*e.g.,* Fig 11). Some technological attributes are summarised in Table 2, which presents the qualitative and quantitative characteristics of flakes and chips ≥10 mm by reduction strategy, including platform type, platform depth, EPA, and number of sharp edges. We emphasise that this classification was not used in the multivariate analysis, where the dataset was constructed solely from complete, unretouched flakes following the attributes listed in Table 1, without applying *a priori* assumptions about the technique. The full dataset is provided in the S3 Appendix. While many flakes align with expected freehand traits, a subset displays features consistent with bipolar percussion, such as linear or broken platforms, flat ventral faces, irregular or absent ripples, and opposed crushing. However, we stress that these traits are not diagnostic exclusively for the bipolar technique. For instance, the absence of ripples is also common in freehand knapping on radiolarite; Siret fractures, typically linked to bipolar, may result from natural cleavage planes (*e.g.,* Fig Ee S1 Appendix); and step terminations, while frequent in bipolar flakes, may also reflect raw material quality (*e.g.,* Fig D S2 Appendix). Conversely, we note the occurrence of feather terminations in flakes that otherwise appear bipolar, a trait not typically associated with bipolar reduction in the literature, further underscoring the material-driven variability. This ambiguity justifies our decision to rely primarily on experimental comparisons and multivariate analysis to investigate reduction techniques for flake production at Marathousa 1.

**Table 2:** Comparison of archaeological flakes and chips (≥10 mm) attributed to bipolar-on-anvil and freehand techniques.

	Bipolar on anvil	Freehand
Flakes and chips (≥10 mm)	24	167
Fragmented	7 (29%)	24 (14%)
Platform types	Linear (7), Broken (4), Flat (4), Natural (3), Punctiform (2)	Flat (55), Faceted (18), Linear (16), Natural (11), Broken/battered (9), Punctiform (2)
Median platform depth	3 mm	4 mm
Median EPA	80°	80°
Mean sharp edges per flake	1 (σ = 0.83)	2 (σ = 0.78)

**Fig 12:**
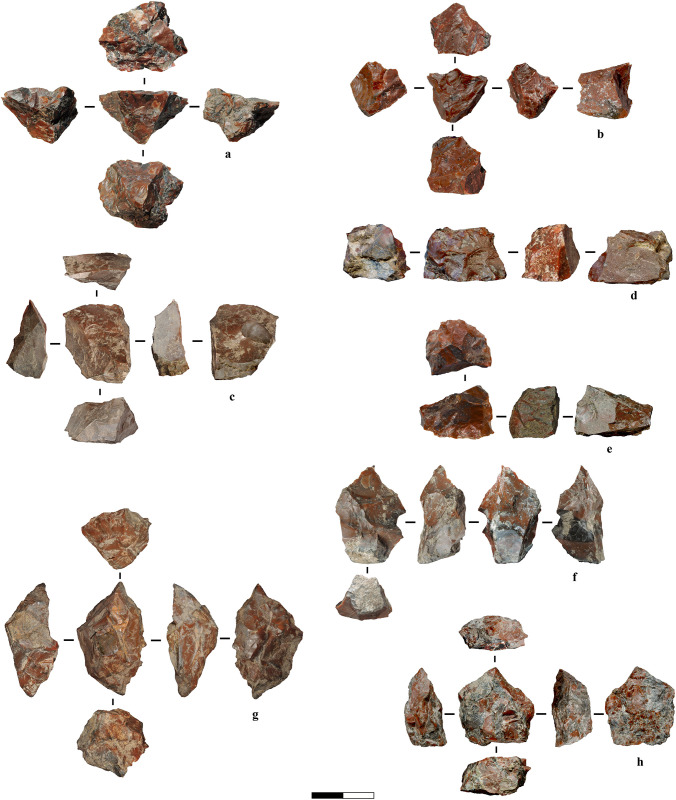
Examples of radiolarite cores. a) Unidirectional sub-pyramidal exhausted freehand core. b) Exhausted multidirectional core. The core exhibits bipolar reduction traces with chips removals in its final phase. c) Core fragment with unipolar removals of flakes produced through freehand technique. It is attributed to the same original reduction sequence as three refitted flakes (Fig 15: 2), although these flakes do not directly refit with the core itself. d) Exhausted bipolar core with multiple crushing traces. The core exhibits irregular removal of chips, possibly as a means of shaping an active edge for use as a tool. e) Exhausted core with multiple flaking surfaces, possibly resulting from freehand reduction. Removals along one side of the core can be related to shaping a functional edge, suggesting a transition from a core to a potential tool. f) Pointed tool on an exhausted multidirectional core with flake and chip removals. The scars are consistent with other chips and flakes found in the assemblage (*e.g.,* Fig 6d). f) Core with a converging morphology at opposite ends, likely resulting from bipolar reduction, with possible subsequent reshaping as a tool. h) Exhausted core with a multidirectional flaking pattern and evidence of bipolar reduction. The pointed morphology results from two opposing flake removals, while a larger flake detachment on the opposite surface has thinned the tip area, possibly enhancing its functionality.

The total number of cores in the assemblage is 29, increasing to 39 when including tools on cores and tested blocks/angular debris. Among this broader group, 34 are made of radiolarite. A total of 16 cores, tested blocks, and tools on cores exhibit clear evidence of bipolar knapping in their final exhaustion stage or, in the case of tools on cores, as part of their final shaping process. In contrast, the remaining cores predominantly exhibit freehand reduction or mixed traces of both techniques. The largest piece associated with bipolar reduction is a limestone tested block, measuring 125 mm x 117 mm x 65 mm, which may have functioned as either a tested core or an anvil. This piece is broken into two refitted sections (see Fig M S1 Appendix), each displaying battering marks on opposing surfaces. The radiolarite cores *stricto sensu*, limited to pieces that exclusively retain core characteristics without further modification, are 25. These cores measure up to 37 mm for bipolar cores and 28 mm for freehand cores. Both freehand and bipolar reduction techniques are linked to multidirectional flaking patterns, though freehand cores also exhibit unidirectional and centripetal flake removals. Many of the identified bipolar cores are small in size, bearing opposing percussion impacts, crushed platforms, stepped terminations, flat surfaces, lack of counter-bulbs and irregular edges possibly resulting from repeated anvil compression. Some cores underwent reshaping, while others were modified through edge retouching. In certain cases, determining whether a core, particularly one with negatives of chips, was fully exhausted remains challenging, as some of these negatives may have been intentionally retained as part of a deliberate shaping strategy (Fig 12).

To summarise, freehand knapping is primarily associated with flake production (*e.g.,* Figs 6, 7: 1–4), whereas bipolar knapping was employed in broader circumstances such as:

- Testing pebbles and during initial decortication (Fig 11).
- Core final reduction, facilitating additional blank detachment from otherwise unworkable material (*e.g.,* Fig 12).
- Flake production, as shown from comparative flake analyses and a refit (Fig 7e, f, Fig 15a, Fig F S1 Appendix).

**Fig 13:**
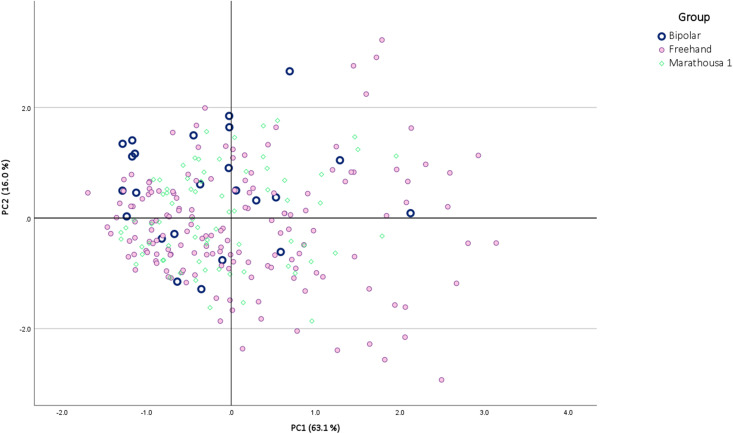
PCA based on linear metric data. The eigenvalues and loadings are listed in Table 3.

**Fig 14:**
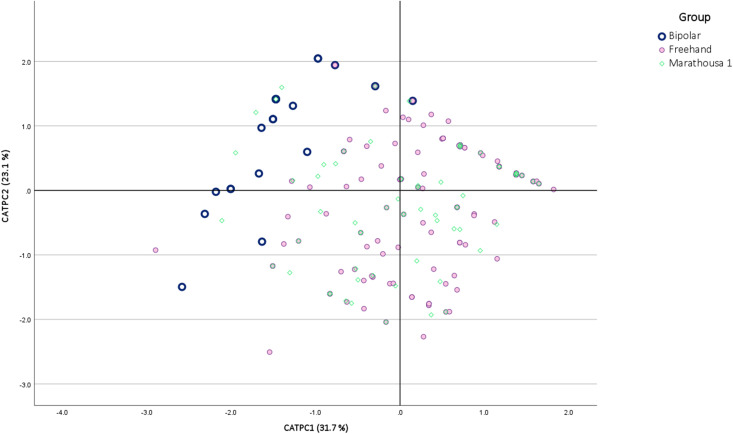
CATPCA based on qualitative flake attributes. The eigenvalues and loadings are listed in Table 4.

### Experiment results and statistical analyses

During the four-day experiment, the bipolar technique was employed in nine full knapping sequences, the freehand technique was used in thirty-one sequences, and the anvil-assisted bipolar technique was employed in one full sequence (used as control). The techniques were combined in another nine sequences. For the multivariate analyses, we utilised 24 bipolar flakes and 154 freehand flakes. For more explicit technical details on the experimental materials see the S2 Appendix on Experimental Data and Statistics paragraphs.

Two multivariate analyses (PCA and CATPCA) were conducted to assess the primary reduction strategies. The analyses relied on an extensive set of quantitative (PCA) and qualitative (CATPCA) flake characteristics. This set of traits from the experimental products was used to determine how bipolar-on-anvil debitage compares to freehand debitage. The Marathousa 1 lithics (N = 90) were then plotted to assess where they fall in relation to these two techniques, helping to identify which reduction method was most prevalent at the site. In the PCA (based only on linear metric data), the first two PCs represent about 79% of the total variance in the sample (Fig 13). Overall, there is excessive overlapping between the two reduction strategies. Based on the factor loadings (Table 2), variation on PC1 represents differences across flakes in overall size, which tended to be greater in freehand flakes. On the PC2 axis, positive values indicate proportionally longer and thicker flakes, which are more frequently the products of bipolar knapping. In contrast, negative PC2 values are more associated with freehand knapping and consist of relatively wider flakes with proportionally larger and thicker platforms. The Marathousa 1 flakes do not present distinctive clustering within the plot.

In CATPCA (focusing on qualitative flake attributes), the first two CATPCs together represent 54.8% of the total variance in the sample (Fig 14). In this analysis, a much clearer distinction emerges between bipolar and freehand flakes, which is overall driven by a combination of traits associated with flake termination, platform preparation, the presence of a central ridge or crest, sharp edges, and ripples (Table 3). More specifically, bipolar flakes predominantly exhibit negative CATPC1 and positive CATPC2 values. For example, a flake in this direction reflects the co-occurrence of moderate bulb, feather termination, one sharp edge and absence of central convexity, platform preparation and ripples, whereas an extreme bipolar flake in the negative CATPC2 quadrant exhibits flat bulb, stepped termination, no sharp edges, central convexity and thin, close-quartered, non-parallel ripples. Freehand flakes show a combination of negative CATPC2 scores and positive CATPC1 values, representing flakes with single sharp edge morphology, stepped termination, median crest, moderate bulb, and visible parallel ripples. The other PC scores result from various combinations of the above two tendencies. Overall, the majority of the Marathousa 1 flakes overlap with the freehand flakes.

**Table 3:** Statistics of the PCA presented in this study. The factor loadings are represented by length (L), width (W), thickness (T), platform depth (PD), platform width (PW).

Analysis	Component	Eigenvalue	Variance explained (%)	Factor loadings				
L	W	T	PD	PW
PCA	PC1	3.16	63.1	0.72	0.82	0.85	0.82	0.75
	PC2	0.8	16.0	0.59	−0.2	0.31	−0.15	−0.55
	PC3	0.5	9.84	0.12	0.45	−0.99	−0.51	0.06
	Total	–	88.94	–	–	–	–	–

The above observations were additionally supported by Kruskal-Wallis-H tests, which showed significant differences across the three groups in the scores of all PCs (P < 0.01 and H-values ranging between 16.5 and 69.8), except for PC1 of the PCA (P = 0.05). Based on the pairwise Mann-Whitney-U comparisons, all the remaining three PCs showed a significant difference between freehand and bipolar flakes (P < 0.01 and Z-scores ranged between −8.2 and −4.0) while also showing a significant variation between bipolar flakes and the Marathousa 1 archaeological assemblage (P < 0.01 and Z-scores varied between −6.6 and −2.9). Additionally, the “control” group B.A.A.) did not exhibit significant differences from the standard bipolar group across all PCs (P > 0.05 and Z-scores ranged between −1.1 and 1.3), so both bipolar variations yield statistically comparable flakes in terms of metric and qualitative attributes (Tables G-K and Fig C, D in S2 Appendix). In contrast, no significant difference was found between the experimentally produced freehand flakes and the archaeological assemblage of Marathousa 1 across components (P > 0.05 and Z-scores ranged between 0.08 and 0.22) (Tables L-S in S2 Appendix).

Taken together, our interpretation of the coexistence of freehand and bipolar knapping at Marathousa 1 is supported not by any single line of evidence but by the convergence of technological observations, experimental results, and multivariate analyses, all grounded in established methodological approaches.

**Table 4:** Statistics of the CATPCA presented in this study. The factor loadings are represented by the bulb of percussion (BP), flake termination (FT), sharp edges (SE), central crest/ridge (CC), platform preparation/trimming (PP), and ripples (R).

Analysis	Component	Eigenvalue	Variance explained (%)	Factor loadings					
BP	FT	SE	CC	PP	R
CAT	CATPC1	1.93	32.12	0.66	−0.4	0.65	0.34	0.62	0.63
PCA									
	CATPC2	1.39	23.11	−0.3	−0.8	0.6	−0.11	−0.3	−0.45
	CATPC3	1.03	17.2	−0.4	0.8	0	0.84	0.3	−0.28
	Total	–	72.42	–	–	–	–	–	–

### Raw materials and technology at Marathousa 1

A total of 53 radiolarite RMUs were identified, comprising 424 pieces (78% of the sample), while another 22 RMUs correspond to other raw materials (N = 87 pieces). The distribution of RMUs for non-radiolarite materials includes 10 limestone RMUs, 5 flint RMUs, and 7 quartz RMUs. The remaining pieces do not cluster into any multi-artifact RMU and are thus considered “isolated RMUs”; among these are flakes made of sandstone and mudstone. Raw material procurement primarily focused on secondary deposits, particularly for radiolarite and limestone, while the sources of flint and quartz are less well-defined.

Radiolarite in the Megalopolis Basin is highly tectonised and, on average, of medium-to-low quality, often characterised by internal fractures, cleavage planes, and variable knappability. Primary outcrops are located at least 4.4 km away from the site, such as those in the bedded Jurassic Formation of the Pindos. Material from these sources is highly brittle, often containing calcite veins, saccharoidal textures, and cleavage planes that hinder conchoidal fracture, making it virtually unknappable. Furthermore, these outcrops are embedded in solid rock formations or large detached boulders, making direct extraction impractical. Even when fractures occur, the resulting material is generally unsuitable for knapping, and no specimens from these outcrops are present in the archaeological assemblage.

In contrast, secondary deposits provided a more accessible source of radiolarite, where water-transport processes not only facilitated availability and provision but also enhanced knappability. These sub-angular pebbles were sourced from riverbeds, streambeds, and colluvial contexts near the site, the closest being ~2 km away. Continuous abrasion and mechanical sorting during transport removed the outer, more brittle fragments, leaving pebbles with a more homogeneous and consolidated internal structure, making them better suited for conchoidal fracture and knapping. However, despite this improvement, the internal quality of radiolarite pebbles remains highly variable and not always predictable from external appearance. Some pebbles, though seemingly homogeneous, contain oxidised internal structures (Fig J S1 Appendix), leading to low hardness making the material too fragile for effective flake production. Others include quartz veins and cleavage planes, which, at best, allow for the production of small flakes with step terminations, as fracture propagation is disrupted by internal discontinuities. In contrast, some pebbles are almost entirely or fully knappable, but due to this variability, testing at the procurement site was likely necessary to avoid transporting unusable material. The same variation in internal quality is also evident in the archaeological assemblage, which includes radiolarite of differing knappability, from high-quality specimens to more challenging materials. However, while secondary deposits contain both knappable and unknappable pieces, only the former are present in the archaeological assemblage, suggesting a selective approach.

Limestone was likely procured from secondary deposits, namely limestone pebbles that co-occur with radiolarite in streams and colluvial outcrops. The nearest primary sources are located 3.84 km west (Cretaceous limestone) and 10 km east of the site (Neogene deposits of the Trifolo Formation). The limestone raw material units (RMUs) in the assemblage include flakes, tools, debris, cores, a tested block, and a hammerstone, with artefacts larger on average than those made of radiolarite and flint.

Flint is found sporadically in secondary deposits; in primary sources, it is usually embedded within limestone formations. It occurs in black and greenish varieties in a streambed 6.80 km away from the site, and in grey flint occurrences 10 km to the east, associated with the Elisson River. RMU analysis indicates that flint was mainly transported as blanks to be retouched (or reshaped) on-site. This conclusion is supported by the presence of chips and microchips from the same RMU, with no evidence of initial core reduction occurring on-site (*e.g.,* Fig Ga, b, e, f, h S1 Appendix).

Quartz pebbles were possibly sourced from the Alfios River valley (~8.6 km away). RMU analysis shows that some quartz was introduced as blanks, but evidence of on-site knapping, mainly through bipolar reduction, indicates local processing, too. Quartz RMUs represent the reduction stages of initialisation, production, and discard of residual cores (Fig N S1 Appendix). Currently, no primary quartz sources have been identified within the basin.

The analysis of RMUs suggests that the *chaînes opératoires* represented at the site are occasionally fragmentary, with some phases, such as early core reduction or final discard, likely occurring off-site. In addition, the RMUs point to variability in reduction strategies and possible core exploitation patterns.

**Fig 15:**
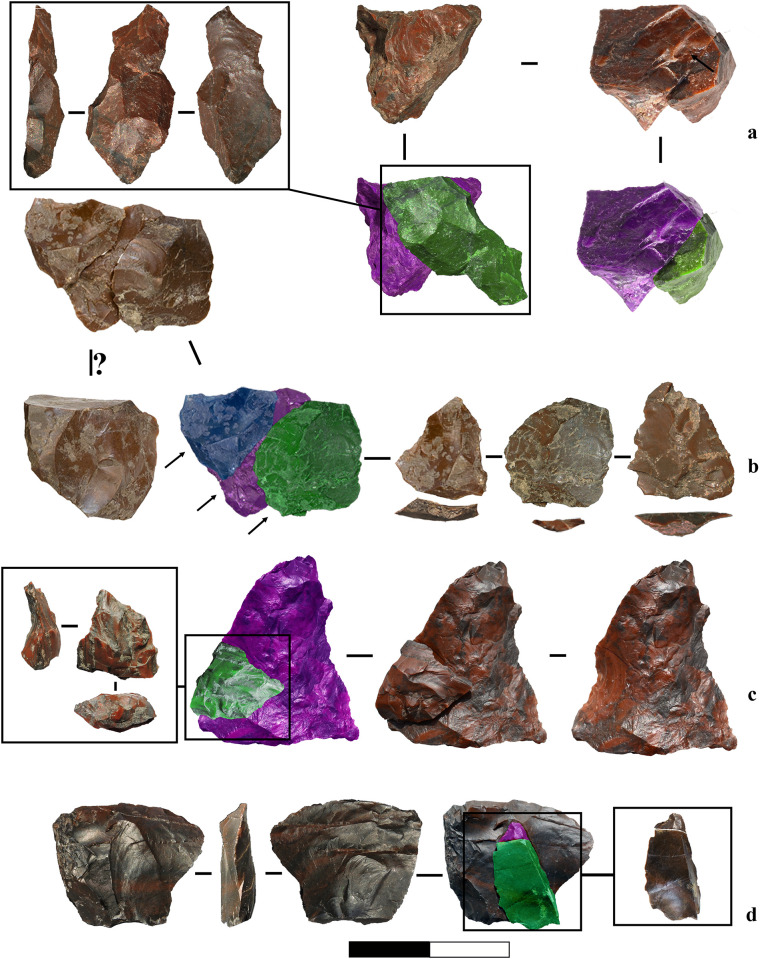
Marathousa 1, refits and conjoins. a) Refit of an elongated flake with a flat ventral surface and exhausted core, both associable with bipolar reduction. b) Refit of three unidirectional small flakes, obtained through freehand percussion, likely belonging to the core fragment of the same RMU. c) Refit of a pointed flake with an orthogonal small flake removal. The latter functions as a confection flake, intentionally detached to create a notch on the main piece. d) Refits of an orthogonal chip (with conjoin) and a flake, possibly detached for thinning or convexity management.

These interpretations are cautiously supported by the refitting results, which provide limited but informative indications into core management and flake removal dynamics. More specifically:

- A refit of an elongated, off-axis flake with a flat ventral surface and a small, exhausted core illustrates the progression of core reduction. The flake exhibits multidirectional scar patterns without counter bulbs and suggests a bipolar reduction sequence involving multiple core rotations. (Fig 15a).
- A refit of three unidirectional small flakes shows a sequence of extractions within freehand reduction. (Fig 15b).
- A small orthogonally removed flake from a larger flake suggests dual functionality, serving as both a confection flake for shaping and a cutting tool (Fig 15c). This aligns with use-wear evidence (see Fig 6d), where similar utilised target flakes share comparable removal patterns with exhausted cores/tools-on-cores (Fig 12f).
- A refit of an orthogonal chip and a flake, possibly detached for convexity management or thinning, may reflect some degree of core preparation awareness. (Fig 15d).
- Additionally, a refit of a limestone block reveals few traces of battering, and the removal position suggests a type of percussion involving an anvil. This artefact may represent a tested block opened through the bipolar-on-anvil technique and subsequently discarded. Alternatively, given its flat morphology, it could have served as an anvil itself, later broken during use.

## Discussion

### Technological behaviour and dynamics in Marathousa 1

The lithic industry at Marathousa 1 provides new insights into Middle Pleistocene hominin technological behaviours in a region crucial for human dispersals during the Lower Palaeolithic [21]. The diversity in raw materials and knapping strategies reflects a technological system suited to balancing efficiency, material economy, and functional demands. The *chaîne opératoire* at Marathousa 1 are mainly focused on local radiolarite. Selection, testing, and partial decortication occurred off-site, as supported by RMU analysis, which shows only some portions of cortical pebbles on-site (*e.g.,* Fig 11). Some of these cortical blanks were later transformed into tools through secondary modification or used as naturally backed knives (see Figs 8, 9). The full reduction sequence focused on producing unstandardised flakes from minimally prepared cores, while tool production followed multiple strategies, including the retouching or reshaping of cortical blanks and the selection of naturally backed knives or unretouched flakes for immediate use. Some tools exhibit steep retouching, creating backed knives with a thick handling/prehensile edge opposite a sharp cutting edge, while scrapers and borers may indicate diverse functional applications. Cores were also occasionally transformed into tools. Beyond reduction-based tools, naturally tabular or subangular blanks were selected and modified through edge shaping and retouching. These naturally occurring pieces, not derived from debitage, were adjusted by enhancing prehensile and functional areas, with their edges worked to create effective cutting, scraping, or perforating implements (for the retouched tools, see Figs 8–10).

The *chaîne opératoire*, encompassing raw material procurement to the discard of cores and tools, appears to be geographically confined within the Megalopolis Basin. The discontinuous pattern of lithic remains, a trait not unexpected in early Palaeolithic records [77], likely reflects a combination of off-site hominin technological activities and post-depositional site disturbances, including recent mining activities. Marathousa 1 lies on the edge of a lignite mine, and while the total extent of the site remains unknown, a significant portion may have been destroyed by mining operations.

The modality of lithic production at Marathousa 1 involved both freehand and bipolar techniques, possibly including the B.A.A. variant, since no significant differences were found within these two techniques, suggesting a flexible approach to core exploitation rather than a rigid reduction scheme. As observed in the assemblage, freehand reduction provided greater control over flake production, while bipolar knapping maximised the exploitation of small or irregular pebbles, increasing raw material consumption. The combination of core management and reduction approaches suggests a functional and effective level of technical know-how [78].

Technology at Marathousa 1 prioritised the maintenance of sharp cutting edges, while also enabling the manufacture of other functional tools, which likely served additional subsistence needs. In an ecological setting such as that of Marathousa 1, a consistent supply of sharp-edged flakes, such as the ones discovered at the site, likely played a key role in processing large animal carcasses. Experimental data [79] indicate that radiolarite flakes dull within approximately 30 minutes of use, reinforcing the need for frequent blank replacement during butchery. Given the fracture characteristics of local radiolarite and the technical proficiency involved, as indicated by the “Persistence” parameter introduced in this study (S2 Appendix), freehand percussion was likely preferred, being faster and slightly more effective for producing sharp-edged flakes.

The multivariate analyses (PCA and CATPCA) highlight the nuanced differences between these two knapping techniques regarding flake morphology. While bipolar flakes are often considered expedient, our study demonstrates that they do not inherently lack sharpness, nor does freehand percussion always result in a flake with feather termination. These variations are influenced by the technique and the fracture properties of local radiolarite, which often produces step terminations or Siret fractures, features less common in fine-grained flint used in prior experimental studies (*e.g.,* [50]).

Among the less-used raw materials, limestone is well represented in the Megalopolis Basin at primary outcrops. While many limestone flakes were expediently produced, the assemblage also contains more complex products, such as a limestone elongated flake (Fig 7d) which bears technological similarities to “non-Levallois” pointed specimens from the Mousterian site of Nahal Mahanayeem Outlet (NMO) [80]. Furthermore, limestone was used to produce robust flakes, tools and hammerstones, possibly introduced as finished products, suggesting that knapping may have occurred near procurement sites, though the presence of a few limestone pebbles/cobbles raises the possibility of on-site reduction or use as hammerstones and/or anvils.

Flint, in black, green, and grey varieties, is present especially in small sizes likely due to its natural fracture properties, in outcrops and secondary deposits within the Megalopolis Basin, which contain numerous cleavage planes (Fig K, L in S1 Appendix). However, the discovery of two larger black flint artefacts in 2013 (Fig 7e, f) suggests that some high-quality flint was transported to the site, though its precise source remains unidentified despite intensive raw material surveys.

Quartz is only sporadically represented within the assemblage. Its reduction appears expedient, possibly limited to situations when other, more suitable materials were not readily available.

The multilayered analytical approach applied to the Marathousa 1 lithic assemblage reveals a structured yet flexible technological strategy. This approach dynamically integrates raw material selection, reduction methods, and tool production according to material availability and possibly functional demands. Within this framework, curated and expedient technological behaviours complement each other, effectively balancing tool efficiency and functional versatility.

### Marathousa 1 in the Eurasian context of the Middle Pleistocene

The role of small tools in the Middle Pleistocene has often been overshadowed by the emphasis on large cutting tools (LCTs) in Lower Palaeolithic research [81]. In 2003, Burdukiewicz and Ronen [20] provided an in-depth analysis of this category, defining small tools as blanks with an average length of 17–35 mm and highlighting their extensive but non-uniform distribution across Eurasia.

In Greece, Marathousa 1 stands alone as the only Middle Pleistocene site with small tools. The only other site with excavated Lower Palaeolithic material, Rodafnidia on Lesbos (MIS 6 – MIS 8), differs significantly due to the presence of LCTs and Levallois products [82]. Outside Greece, during MIS 15−9 (corresponding to ~621−300 ka) [83], several European sites exhibited technological and contextual affinities with Marathousa 1. In Central and Eastern Europe, sites such as Trzebnica 2 and Rusko 42 (Poland, ~ 700−400 ka) show a clear trend toward microlithisation, with an emphasis on heavily retouched tools [84,85]. At Vértesszőlős (Hungary, ~ 400 ka) small tool production focused on naturally backed knives, primarily made from jasper and radiolarite, while residual cores were either reshaped into tools or left as they were [86–89], characteristics that are also observed at Marathousa 1. In Germany, sites such as Bilzingsleben (~300 ka) and Schöningen (~300 ka) feature both elephant remains and small tool industries, which include expedient artefacts that are often manufactured on natural blanks [90–93]. In Italy, Middle Pleistocene sites frequently associate small tool industries with elephant exploitation. At Isernia la Pineta (~600 ka), the lithic assemblage includes unstandardised and heavily retouched tools, such as denticulates, scrapers, and notches with serrated retouch [94,95], closely resembling elements of Marathousa 1. At Cimitero di Atella (~550 ka), small tools were produced from debris, flakes, and residual core fragments, while unretouched flakes exhibit orthogonal removals designed to create central convexities [87,96,97], strategies that are also observed at Marathousa 1. At Ficoncella (~550 ka), retouch was integral to blank shaping, making the latter difficult to distinguish from tool modification [98–100], a pattern partially reflected in the Marathousa 1 blanks and cores [24]. At Fontana Ranuccio (~400 ka), chert and quartzite pebbles were worked using freehand and bipolar techniques, showing a diverse approach to lithic exploitation, such as in Marathousa 1 [101]. Finally, La Polledrara di Cecanibbio (~340 ka) is a well-preserved butchery site where small flint pebbles were used to produce lithic artefacts, found in direct association with *Palaeoloxodon antiquus* remains within a paleo-fluvial setting [102,103], further reinforcing the connection between small tool production and elephant processing. According to Derevianko [104], hominins using small tool industries were among the first groups to migrate out of Africa via the Near East corridor. This trend is evident in sites like Bizat Ruhama (1.6–1.2 Ma) [56], Evron Quarry (800 Ka) [105,106], Gesher Benot Ya’aqov (790 Ka) [81], Qesem Cave (400−200 Ka) and Revadim (500–300/200 Ka) [107,108].

Rather than representing a monolithic tradition, small tool industries in the Middle Pleistocene exhibit significant variability, shaped by local raw materials, functional needs, or environmental contexts. Marathousa 1 shares core traits with the aforementioned small tool sites, yet it stands out for its contextual and technological specificity. Located within the resource-rich paleolake environment of the Megalopolis Basin, it is characterised by stable ecological conditions, abundant in fauna, flora, and raw materials, particularly radiolarite [4,11,13,15–17,109]. The lithic industry at Marathousa 1 demonstrates considerable adaptability and strategic flexibility. While local radiolarite was predominantly exploited, occasional artefacts made from limestone, flint, quartz (and also isolated ones in sandstone and mudstone) were also introduced, all exhibiting a distinct microlithic trend toward small flakes, with rare exceptions such as a large flint flake (Fig B S1 Appendix). Unlike other small tool sites, Marathousa 1 exhibits a strategic interplay of bipolar and freehand knapping techniques that are adjusted according to the stage in the *chaîne opératoire*. While the bipolar technique generally played a secondary role, it was required in specific contexts, notably during initial core testing phases and late-stage exploitation, when freehand knapping would become less viable. The assemblage predominantly consists of simple flakes, including naturally backed knives for immediate use without further modification. Experimentally, it has been demonstrated that these small radiolarite flakes are highly effective in butchery tasks [79], challenging traditional assumptions favouring larger flakes [110]. A secondary modification was also selectively applied to create scrapers, denticulates, and borers, alongside tools shaped directly from natural blanks and cores. Furthermore, the EPA and platform depth of the flakes suggest similarities with younger lithic assemblages in terms of flake sharpness (Fig 2); this becomes even more apparent when considering only those flakes attributed to freehand knapping. Finally, Marathousa 1 preserves a direct association between small tools and remains of *Palaeoloxodon antiquus,* clearly indicating carcass processing as one of the most significant hominin activities performed at the shores of the Megalopolis paleolake.

Regarding the latter point, a feature sometimes present among small tool sites is the exploitation of elephants, described as an ‘organic quarry’ by Lemorini (2018) [111], providing both food and raw materials, a practice evident from the Early Pleistocene (~2 Ma, Africa) until their extinction in the Late Pleistocene [102,103,112–119]. At Marathousa 1, *Palaeoloxodon antiquus* similarly played a central role, as indicated also by the close spatial association of its remains with lithic artefacts. Evidence of anthropogenic modification, including a denticulated bone flake and a bone percussor [24], highlights the role of megafaunal remains not only for subsistence as inferred by cutmarks on bones [13], but also for tool production. Comparable patterns are also documented throughout the Pleistocene Old World [*e.g.,* 115,120,121], but also among Clovis populations in the New World, where mammoths served as key resources providing immediate sustenance as well as materials for tool manufacture [122].

The significance of small tool industries is closely tied to their remarkable versatility. Use-wear studies provide evidence that flakes smaller than 20 mm were employed in carcass-processing activities, including the butchery of proboscideans [98,102,105,111,112]. However, their utility extended beyond carcass processing, as small flakes were also effectively used for working perishable materials like wood and plants [100,118,120,122-125], a multipurpose role preliminarily documented at Marathousa 1 as well [25].

Concerning how these small tools were used, one of the key debates revolves around whether they were hafted or handheld. Hafting is known to enhance grip and control, particularly in tasks such as hide-working, where exposure to grease, blood, and other lubricants could reduce handling efficiency ( [126] and references therein; [127], p. 55). However, experimental elephant butchery [80], with small flakes, which were produced from the same Megalopolis Basin radiolarite examined here and replicated the Marathousa 1 morphologies, demonstrated that small flakes could be effectively handheld for elephant butchery, securely grasped between two fingers to ensure precision and control. In fact, despite their size, small flakes from multiple sites, including Marathousa 1, exhibit excellent prehension ergonomics [79,120], raising questions about whether hafting was necessary. Fluck [128] notes that while the small size of blanks at Vértesszőlős may have been dictated by raw material constraints, sites like Trzebnica and Rusko suggest a deliberate preference for intentionally miniaturised artefacts. Some researchers interpret this as an indication of advanced manual dexterity in *Homo erectus* [106, p. 360].

At Marathousa 1, certain tools exhibit surface thinning that may have enhanced gripping. Others, produced from natural blanks, feature a combination of actively retouched sections and unmodified areas, suggesting that some pieces were intentionally designed to facilitate handling without requiring hafting. This aligns with broader patterns seen across Middle Pleistocene small tool industries, where direct hand-held use appears to have been a viable and widely adopted strategy (*e.g.*, [105,106]).

## Conclusion

The Marathousa 1 lithic assemblage reflects a pragmatic and versatile technological system, shaped by raw material availability and subsistence needs in the Middle Pleistocene. The emphasis on small blank production, achieved through both freehand and bipolar knapping, indicates a flexible strategy for core exploitation while responding to raw material constraints. The presence of *Palaeoloxodon antiquus* in association with lithic artefacts suggests that megafaunal exploitation played a central role in shaping technological behaviour at the site. The demands of processing such a large carcass likely influenced lithic production, favouring strategies that ensured a reliable supply of flakes, where practical choices took precedence over standardised tool forms. Within the broader Lower Palaeolithic context of small tool sites, Marathousa 1 stands for small flakes produced through the strategic interplay between bipolar and freehand knapping, tailored to local radiolarite quality and fracture properties. However, this focus on flake production was complemented by the presence of highly retouched tools, which may have served additional needs beyond butchery. Their coexistence within the assemblage reflects a strategy that moves beyond the dichotomy of expediency and curation, indicating a dynamic system in which immediate functionality and extended tool use were integrated.

### Permits

All necessary permits were obtained from the Ephoreia of Palaeoanthropology-Speleology (Ministry of Culture and Sports, Greece) for the described study, which complied with all relevant regulations.

## Supporting information

S1 Appendix Archaeological artefacts and geological samples.
(DOCX)

S2 Appendix Experimental data and Statistics.
(DOCX)

S3 Appendix Multivariate analyses.
(XLSX)
