# Supplementary material for: Small flakes for sharp needs: Technological behaviour in the Lower Palaeolithic site of Marathousa 1, Greece
Source: PLoS One. 2025 Jun 30;20(6):e0324958. doi: 10.1371/journal.pone.0324958 (PMC12208439; doi:10.1371/journal.pone.0324958)
Supplement: S1 Appendix — Archaeological artefacts and geological samples. (DOCX) [file pone.0324958.s001.docx]

**S1 Appendix.** Archaeological artefacts and geological samples

# Outlier


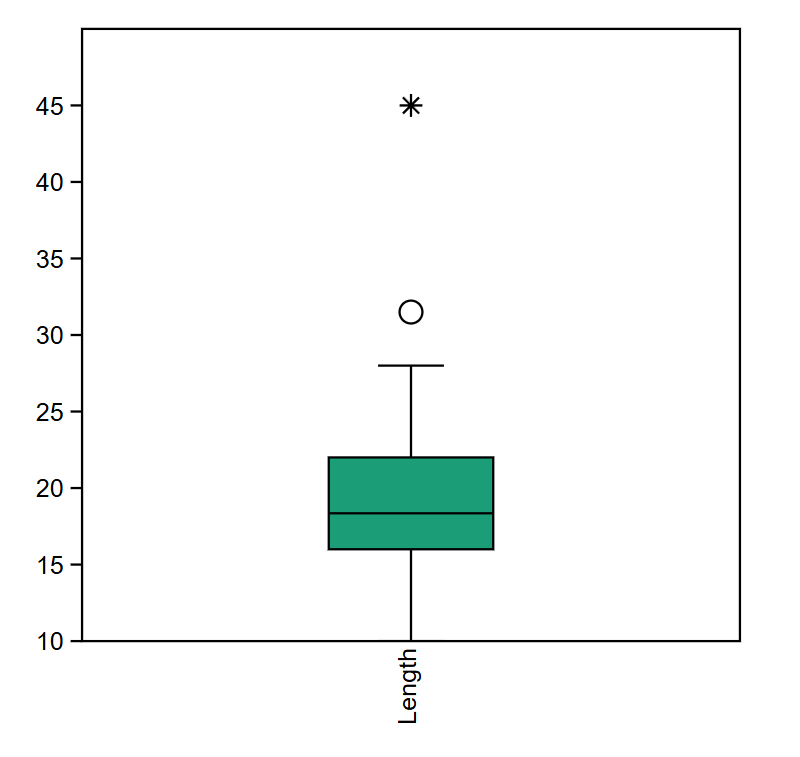


**Fig. A. Boxplot to identify the outlier among radiolarite complete flakes to run statistical analyses.**


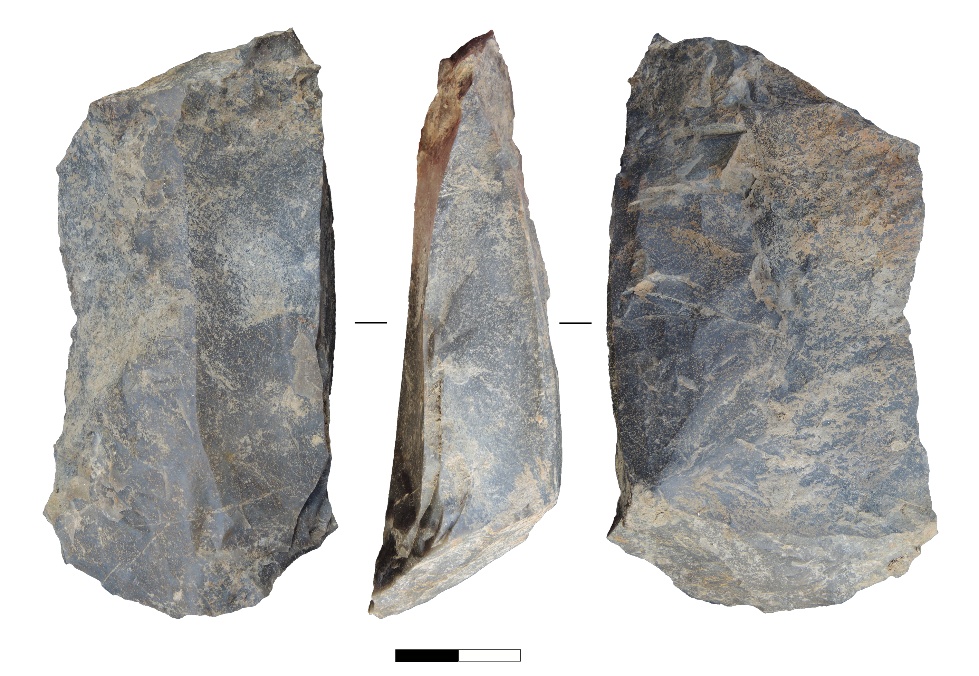


**Fig. B. Flint naturally backed knife. Outlier flake among the whole flake assemblage.**

# Dimensions of artefacts


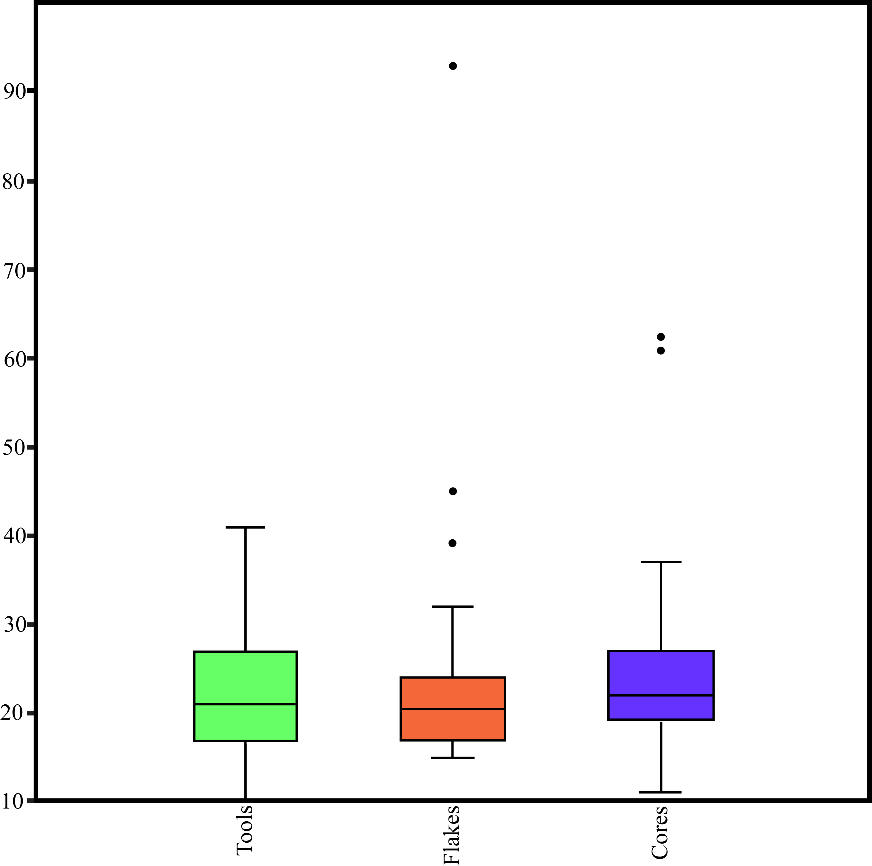


**Fig. C. Boxplots with dimensions of flakes tools and cores of all raw materials.**


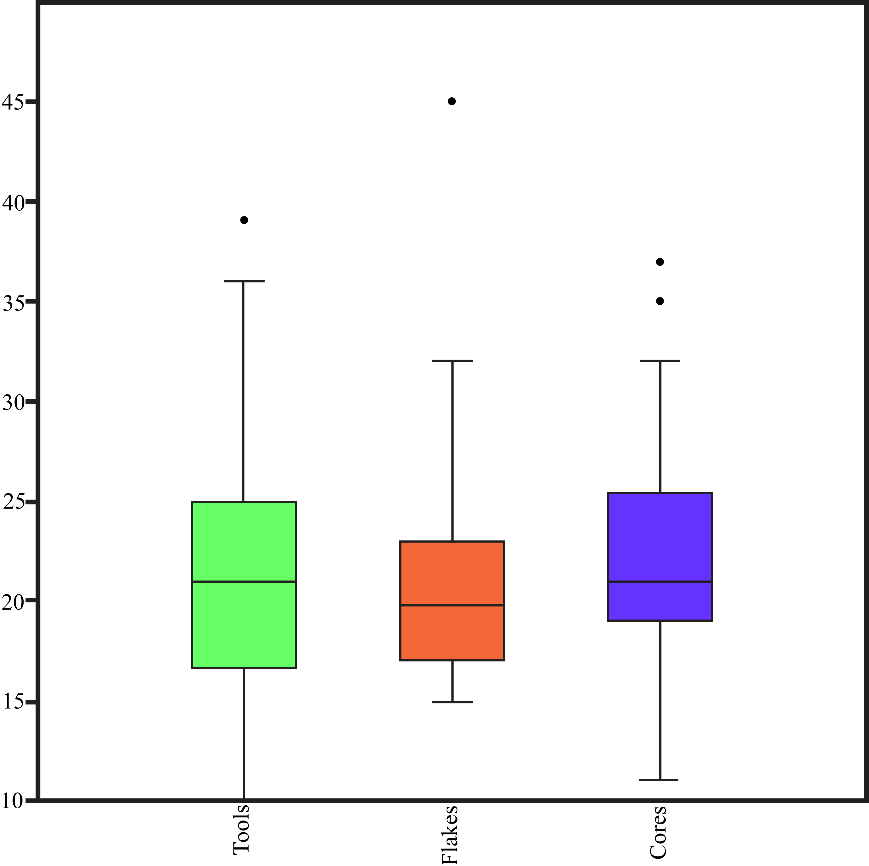


**Fig. D. Boxplots with dimensions of radiolarite tools, unretouched flakes and cores.**

# Flakes


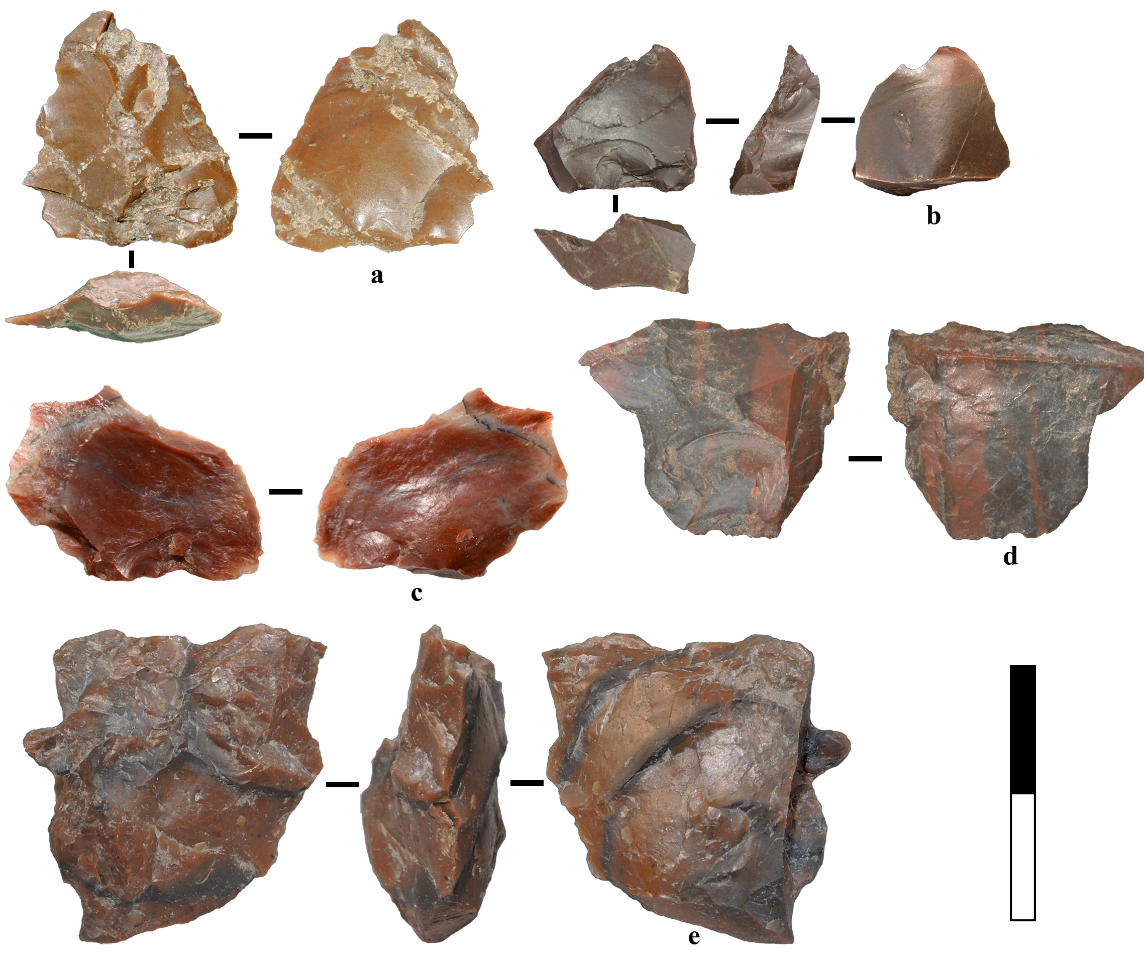


**Fig. E. Freehand radiolarite flakes.**


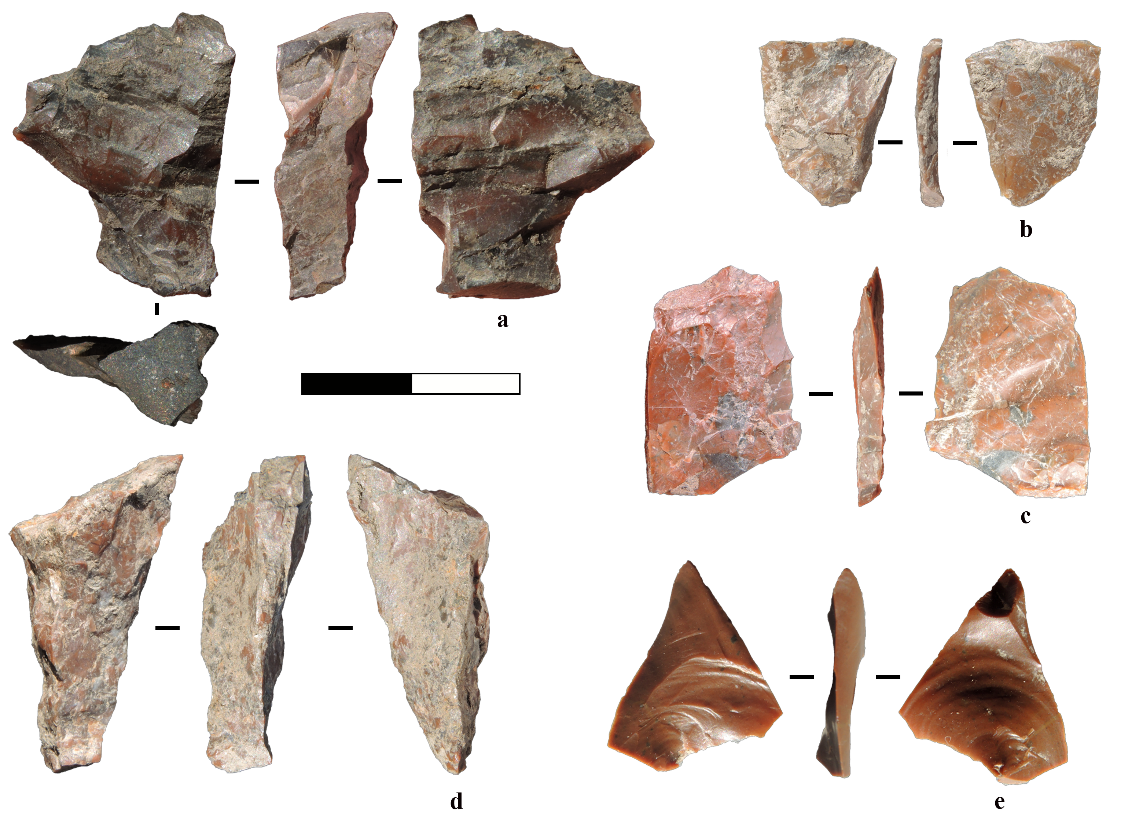


**Fig. F. Bipolar radiolarite flakes.**

# Tools


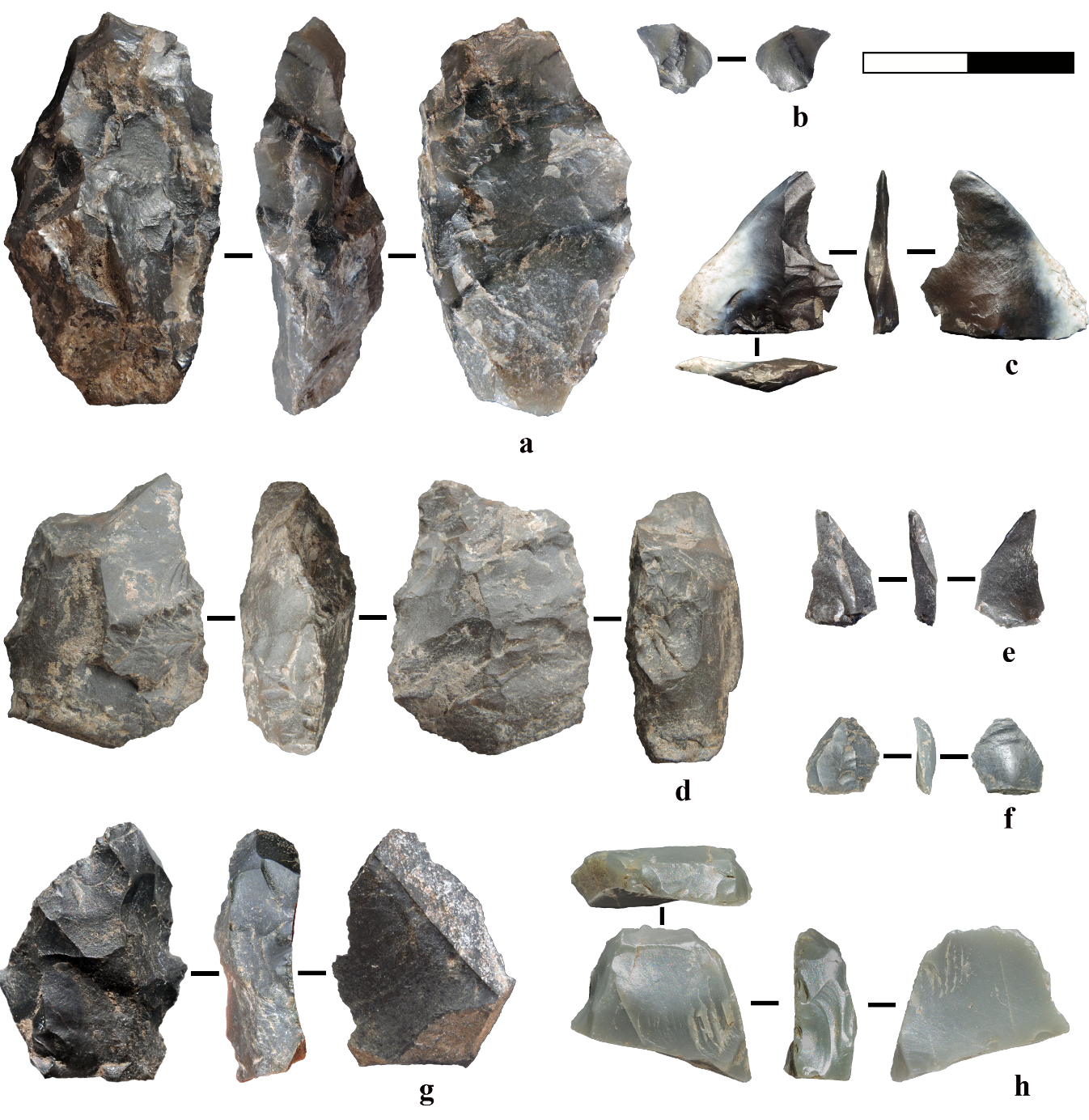


**Fig. G. Tools and retouch chips produced utilising other raw materials.**(b) is a retouch chip produced from (a) (f) is a retouch chip derived from the same RMU as (h).

# Raw materials


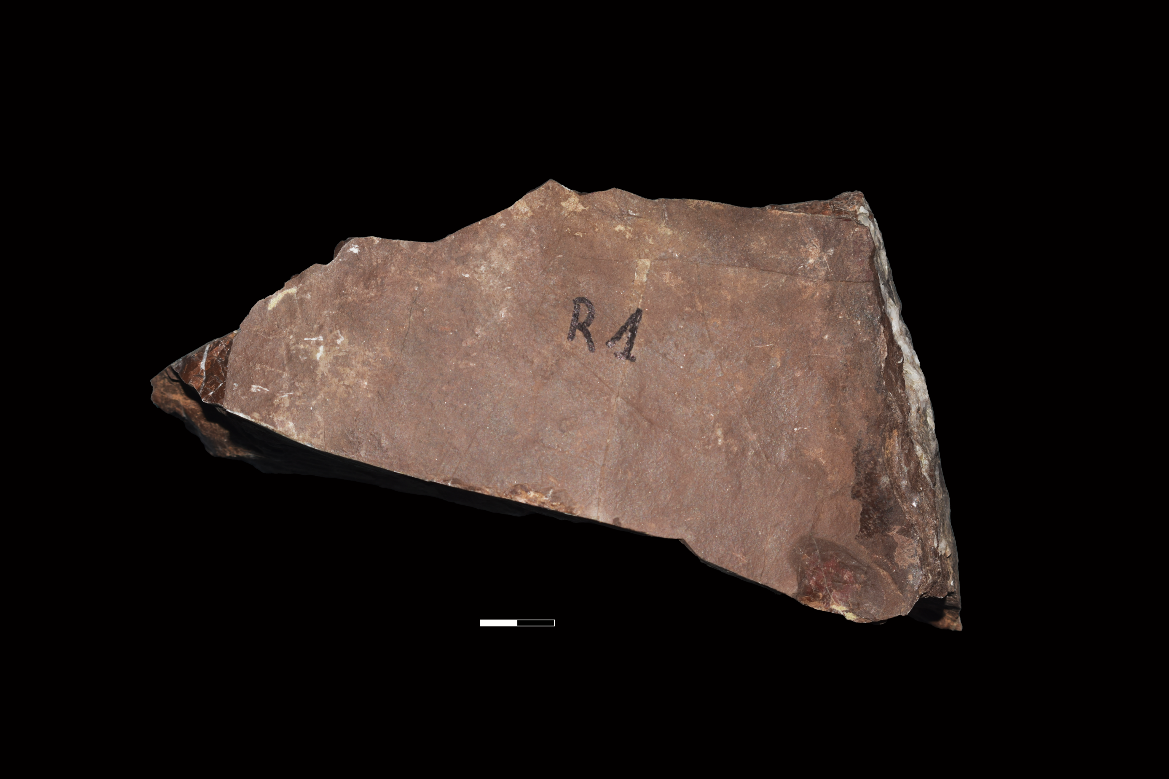


**Fig. H. Radiolarite block from primary deposit in Megalopolis basin.**

**
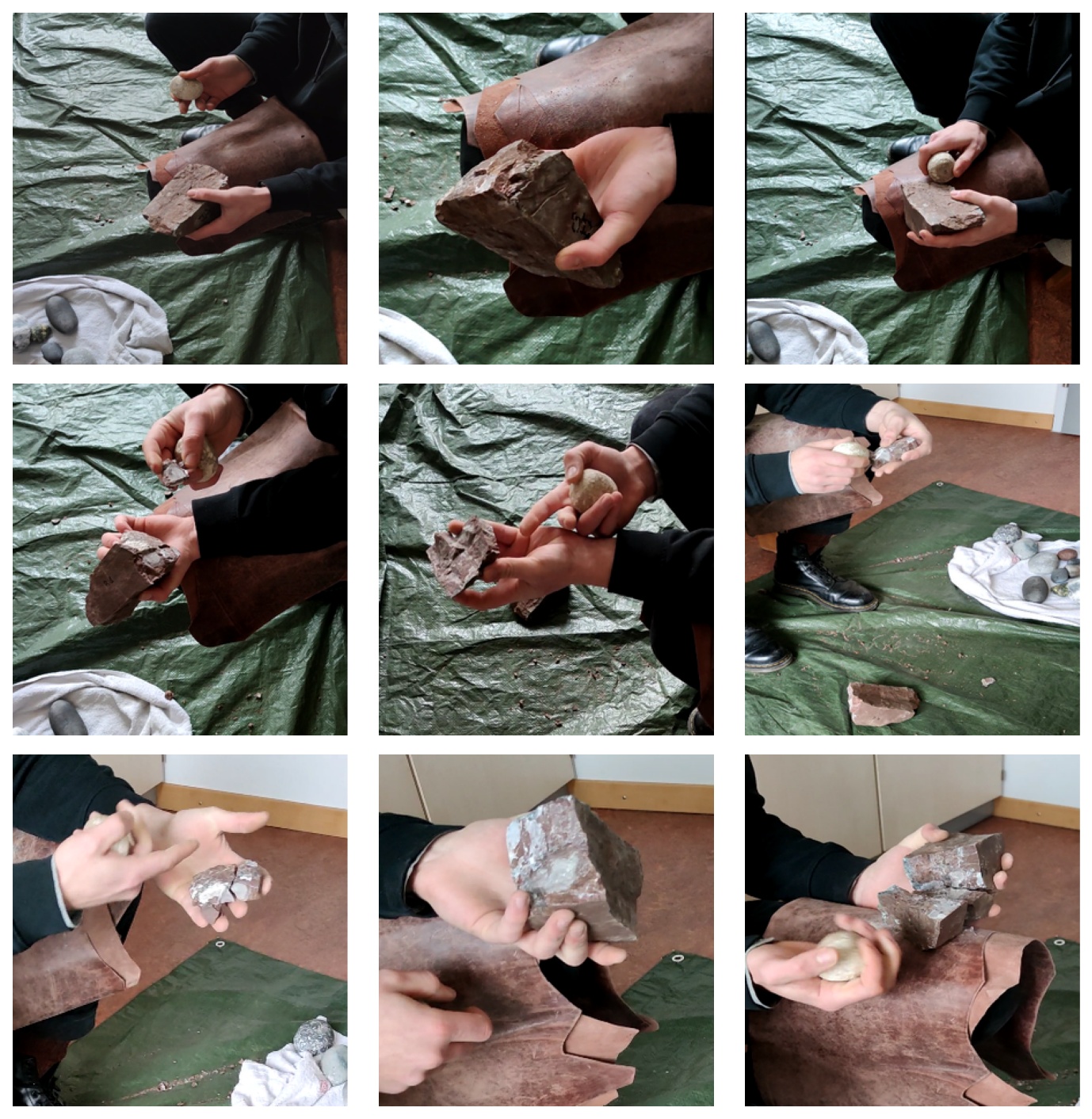
**

**Fig. I. Knapping of a radiolarite block from a primary deposit in the Megalopolis basin.**

Sequential views illustrate the percussion of a core (R1), and the challenges associated with exploiting radiolarite from primary geological sources. Despite initial removals, fracture propagation was irregular due to the internal structure of the piece. The sequence documents early attempts at initiating conchoidal fracture, the formation of irregular breakage surfaces, and the eventual detachment of chunks without anthropogenic features. The core shows oxidation and cleavage planes that hindered effective knapping.


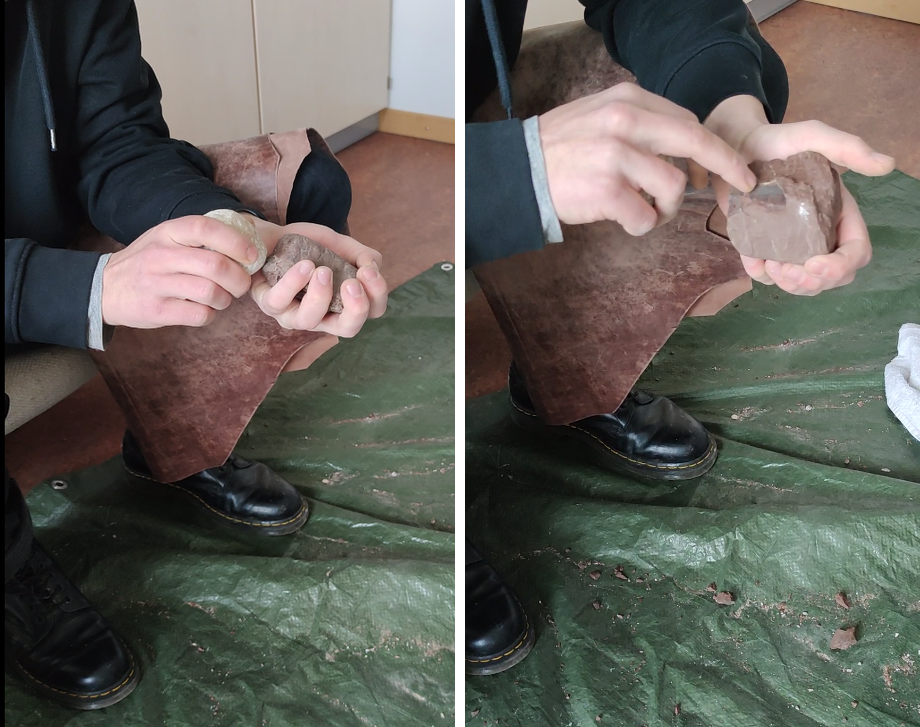


**Fig. J. First knapping attempt on a radiolarite pebble.**

The pebble was selected based on its suitable morphology and apparent external homogeneity. However, the first blow revealed an unexpected internal oxidation zone, which compromised the integrity of the raw material and led to the abandonment of the core.


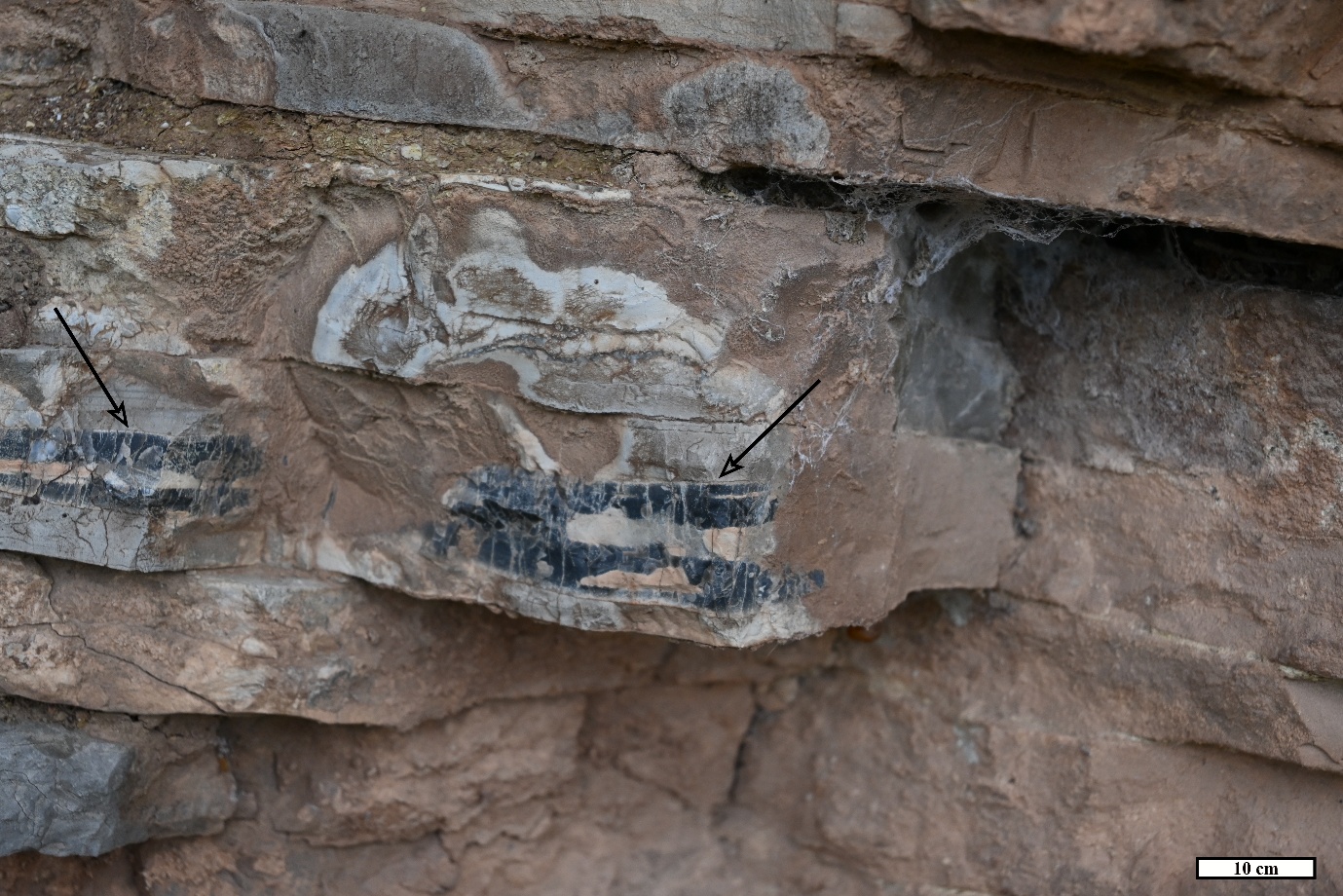


**Fig. K. Black flint in limestone outcrop (Kyparissia).**


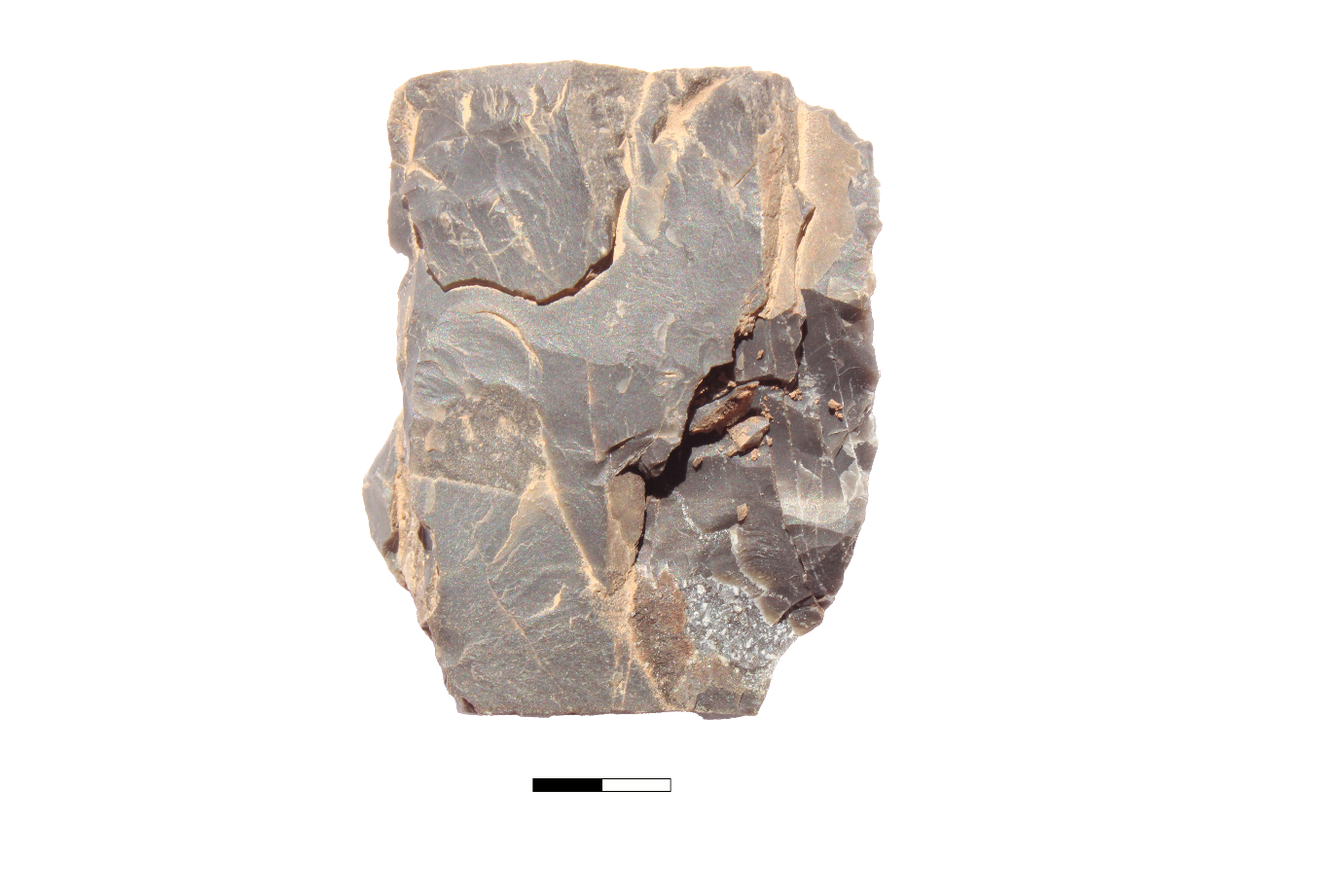


**Fig. L. Dark grey flint collected in a riverbed (Kyparissia).**

**
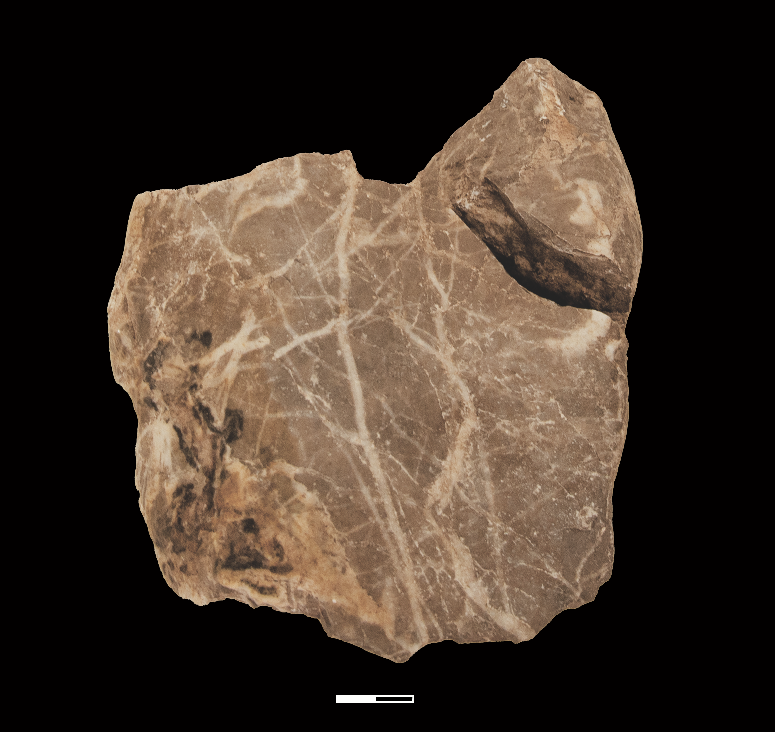
**

**Fig. M. A limestone block with anthropogenic features broken in two pieces.**

**
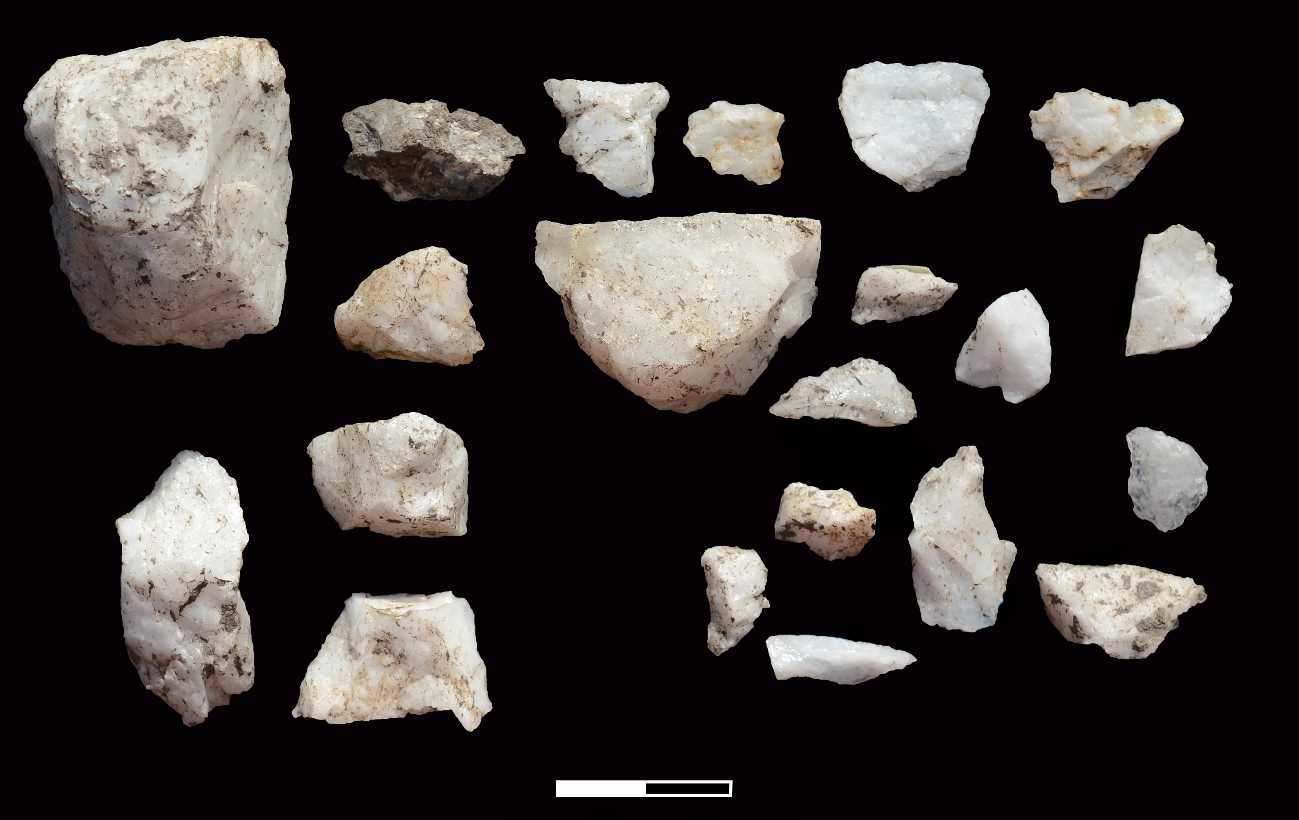
**

**Fig. N. Quartz artefacts.**

# **Essential terminology**

**Blank**: A blank is any element from which an object is knapped, shaped, flaked, or retouched. It includes slabs, chunks, debitage products, and other lithic supports. At Marathousa 1, tools were produced from various types of blanks. When referring to a generic support for tool production, the term 'blank' is used in its broadest sense.

**Debris**: Also defined as 'knapping waste/by-products,' debris refers to a material residue that is not predetermined, retouched, or conceived *a priori* as tool blanks. In this study, these terms are used collectively for all waste material anthropogenically generated during lithic reduction.

**Flake and Target Flake**: A flake is a general term for a fragment of hard stone removed from a core, slab, or other support, with one identifiable ventral face. It may be further shaped into a tool or detached from it during manufacture. At Marathousa 1, flakes smaller than 15 mm are classified as chips, while those smaller than 10 mm are called microchips.

A target flake is a flake intentionally produced through a structured knapping strategy to obtain a functionally viable blank, either for immediate use or further modification.

**Tool**: Any lithic artefact that exhibits evidence of intentional modification (retouch) or clear indications of use, such as edge damage or residue traces. Target flakes with use-wear traces also fall into this broad category. At Marathousa 1, tools originate from various blanks, including flakes, cores, natural (unknapped) pieces, or knapping by-products.

In this study, we adopted a parsimonious approach by using the term 'target flakes' to initially describe small (usable and used) flakes that, along with retouched artefacts, characterise the archaeological assemblage associated with the Small Tool Industry. In discussions, we use the broader term 'tools' to collectively refer to Small Tool Industry components.

## **Bibliography**

Mesfin I, Texier P-J. (2022). Prepared core technology from the Early Pleistocene site of Nyabusosi 18, Uganda. *J Archaeol Sci Rep.* 46:103695. doi:10.1016/j.jasrep.2022.103695.

Tourloukis V, Thompson N, Panagopoulou E, Giusti D, Konidaris G, Harvati K (2018). Lithic artifacts and bone tools from the Lower Palaeolithic site Marathousa 1, Megalopolis, Greece: Preliminary results. *Quaternary International*, 497: 47-64. <https://doi.org/10.1016/j.quaint.2018.05.043>

White M, Ashton N, Scott B. (2011). The Emergence, Diversity and Significance of Mode 3 (Prepared Core) Technologies. In: Ashton N, Lewis SG, Stringer C, editors. *Developments in Quaternary Science.* Vol. 14. Elsevier. pp. 53-65. doi:10.1016/B978-0-444-53597-9.00005-4.

Ballin TB. (2000). Classification and description of lithic artefacts: A discussion of the basic lithic terminology. *Lithics.* 21: 9–15.

Inizan ML, Roche H, Tixier J (1999). Technology of knapped stone: followed by a multilingual vocabulary, Arabic, English, French, German, Greek, Italian, Russian, Spanish.

Shott MJ. (1994). Size and form in the analysis of flake debris: Review and recent approaches. *J Archaeol Method Theory.* 1(1): 69-110.
