# Supplementary material for: Small flakes for sharp needs: Technological behaviour in the Lower Palaeolithic site of Marathousa 1, Greece
Source: PLoS One. 2025 Jun 30;20(6):e0324958. doi: 10.1371/journal.pone.0324958 (PMC12208439; doi:10.1371/journal.pone.0324958)
Supplement: S2 Appendix — Experimental data and Statistics. (DOCX) [file pone.0324958.s002.docx]

**S2 Appendix**. Experimental data and Statistics

During the four-day experiment, a total of 11.43 Kg of radiolarite was knapped. The bipolar technique was employed in nine full knapping sequences, while the freehand technique was used thirty-one times. Both techniques were combined in nine instances. Most cores were knapped longitudinally, centred and balanced, ensuring full compression along their long axis. In a single instance, a core was positioned transversally while maintaining full contact with the anvil. The resulting flakes displayed no significant differences in attributes compared to those produced through longitudinal bipolar strikes. In some instances, flakes detached from the surface in contact with the anvil, likely due to counterstrike effects. These flakes were documented, but since they did not exhibit clear technological differences from standard bipolar flakes, they were categorised using the same attribute system. As outlined in the methods the variation in the number of flakes is largely attributed to the semi-autonomy granted to the five volunteers that in many instances found the original core shape to be more suitable for freehand technique than for bipolar.

When considering the ratio (N) of flakes per volume, the success rates for producing flakes of 15 mm or larger were as follows:

- 4% for the freehand technique (264 flakes/6638.5 cm^3^)
- 2.6% for the bipolar technique (39 flakes/1506.7 cm^3^).
- 2.1% for the bipolar anvil-assisted technique (18/861.6 cm^3^).

The average dimensions and mass of the complete experimental flakes were:

- For the freehand technique: 25.27 mm x 19.52 mm x 6.95 mm with a mean mass of 6.27 g
- For the bipolar technique: 21.5 mm x 13.7 mm x 5.7 mm with a mass of 2.8 g.
- For the bipolar anvil-assisted technique: 21.2 mm x 11.7 mm x 5.5 mm with a mass of 2 g.

When present, the platform for freehand flakes is either natural or on cleavage (44%), flat (25%), linear (23%), faceted (6%), or punctiform (2%). These have a median platform depth of 4 mm and an External Platform Angle (EPA) of 80°.

In the case of bipolar flakes, 55.5% have a linear platform, 22% are natural, 18.5% are punctiform, and there is one instance of a flat platform. The median platform measurement is 3 mm with a 90° EPA.

Bipolar anvil-assisted knapping results in 64% linear platforms, with both punctiform and natural platforms accounting for 2% each, and one instance of a flat platform. The median platform depth is 2 mm with a 90° EPA.

The ratio of sharp edges to the number of artefacts (flakes and chips ≧ 10mm) for each applied knapping technique is as follows:

- Freehand: 2.15 (σ 0.77)
- Bipolar: 1.84 (σ 0.80)
- Bipolar anvil-assisted: 2.06 (σ 1.03).

The "Persistence" parameter denotes the number of unsuccessful blows preceding the production of a flake. This is elucidated by the ratio of blows to the number of flakes obtained through each technique. For bipolar knapping, including anvil-assisted methods, this ratio stands at 14.01 (or 8.21 for axial bipolar technique), while for freehand knapping, it is considerably lower at 2.78. Interestingly, when considering only the expert knapper, the blown shots drop further, with ratios of 0.33 for bipolar and 1.35 for freehand techniques.

In the experiment, most freehand sequences were conducted by the highly experienced knapper, while bipolar sequences were largely carried out by participants with less practical experience. While this slight imbalance may have influenced the relative success rates, our aim was not to isolate skill as a variable, but to explore how different techniques perform under conditions that reflect real-world variability in knapper expertise and decision-making. We acknowledge this limitation and interpret the results with appropriate caution, particularly when comparing productivity across techniques.

Regarding core refits for experimental pieces, we selected 13 knapped cores reduced according to the two techniques (2 bipolar, 1 mixed, 10 freehand). Many surfaces remained devoid of anthropogenic features, regardless of the type of technique adopted, and this was a surprising observation, considering the mechanical processes expected. At the macroscopic level, in certain instances, identifying the point of percussion proved to be a daunting task, making it impossible to orient the artefact or distinguish the dorsal surface from the ventral one. Some cases presented a unique scenario where the flake detachment occurred along a parallel plane (cleavage). This unexpected occurrence interrupted the continuation of the ventral surface, transforming it into a natural plane. This phenomenon could be attributed to the inherent mechanical properties of the material and the force vectors applied during the knapping process. A noteworthy observation was that despite employing the bipolar knapping strategy, no flakes with a double bulb were produced, aligning with some findings from the cited literature (Crabtree 1982:16). Similarly, in the archaeological assemblage, no flakes exhibiting a double bulb were identified, further reinforcing this pattern.

The Persistence parameter indicates that the experienced knapper generally required fewer strikes per flake than less-experienced ones. This effect was more noticeable in freehand knapping, where core convexity control played a role. In contrast, bipolar knapping appeared to reduce skill influence. These findings suggest that skill level may have influenced flake counts and size distributions, particularly in freehand experiments. However, both groups of knappers produced an experimental assemblage broadly comparable to the archaeological one, supporting the relevance of the technological interpretations at Marathousa 1.

The average time to refit the cores was 30 minutes, considering that all artefacts and debris from the same core were stored together in one bag and the overall medium to small dimensions of pebbles.

**Table A. Raw material collection localities and measurements.**

| **Code** | **Source** | **Collection Locality** | **Mass (g)** | **Length (mm)** | **Width (mm)** | **Thickness (mm)** |
| --- | --- | --- | --- | --- | --- | --- |
| R-1 | Primary | Karytaina | 3030 | 175 | 90 | 74 |
| R-2 | Secondary | Karytaina | 274 | 130 | 65 | 30 |
| R-3 | Secondary | Karytaina | 468 | 110 | 50 | 63 |
| R-4 | Secondary | Karytaina | 344 | 60 | 49 | 45 |
| R-5 | Secondary | Karytaina | 357 | 90 | 80 | 42 |
| R-6 | Secondary | Karytaina | 804 | 75 | 75 | 55 |
| R-7 | Secondary | Karytaina | 521 | 90 | 60 | 55 |
| R-8 | Secondary | Karytaina | 835 | 102 | 63 | 50 |
| R-9 | Secondary | Karytaina | 663 | 85 | 75 | 40 |
| R-10 | Secondary | Karytaina | 451 | 70 | 62 | 40 |
| R-11 | Secondary | Karytaina | 208 | 50 | 40 | 40 |
| R-12 | Secondary | Karytaina | 327 | 90 | 40 | 35 |
| R-13 | Secondary | Karytaina | 190 | 75 | 50 | 20 |
| R-14 | Secondary | Karytaina | 291 | 80 | 40 | 35 |
| R-15 | Secondary | Karytaina | 182 | 50 | 40 | 35 |
| R-16 | Secondary | Karytaina | 250 | 55 | 50 | 35 |
| R-17 | Secondary | Karytaina | 60 | 65 | 30 | 25 |
| R-18 | Secondary | Karytaina | 175 | 75 | 45 | 20 |
| R-19 | Secondary | Karytaina | 82 | 50 | 30 | 30 |
| R-20 | Secondary | Karytaina | 140 | 60 | 45 | 20 |
| R-21 | Secondary | Karytaina | 172 | 70 | 40 | 30 |
| R-22 | Secondary | Karytaina | 196 | 75 | 55 | 40 |
| R-23 | Secondary | Agios Ioannis | 108 | 42 | 31 | 32 |
| R-24 | Secondary | Agios Ioannis | 34 | 34 | 22 | 12 |
| R-25 | Secondary | Agios Ioannis | 84 | 55 | 31 | 19 |
| F-26 | Secondary | Routsi | 164 | 55 | 36 | 32 |
| R-27 | Secondary | Routsi | 58 | 45 | 25 | 20 |
| R-28 | Secondary | Karytaina | 141 | 55 | 45 | 22 |
| R-29 | Secondary | Karytaina | 400 | 70 | 55 | 40 |
| R-30 | Secondary | Karytaina | 148 | 65 | 50 | 45 |
| R-31 | Secondary | Karytaina | 125 | 60 | 47 | 40 |
| R-32 | Secondary | Karytaina | 321 | 85 | 40 | 75 |
| R-33 | Secondary | Karvounari | 277 | 83 | 65 | 45 |
| R-34 | Secondary | Karvounari | 350 | 85 | 57 | 40 |
| R-35 | Secondary | Karvounari | 816 | 93 | 75 | 45 |
| R-36 | Secondary | Karvounari | 369 | 70 | 58 | 35 |
| R-37 | Secondary | Karvounari | 63 | 55 | 37 | 40 |
| R-38 | Secondary | Karvounari | 204 | 70 | 45 | 25 |
| R-39 | Secondary | Karvounari | 241 | 65 | 65 | 22 |
| R-40 | Secondary | Karvounari | 166 | 55 | 53 | 22 |
| R-41 | Secondary | Karvounari | 351 | 60 | 45 | 50 |
| R-42 | Secondary | Karvounari | 194 | 52 | 45 | 32 |
| R-43 | Secondary | Karvounari | 274 | 60 | 55 | 32 |
| R-44 | Secondary | Karvounari | 773 | 80 | 60 | 62 |
| R-45 | Secondary | Karvounari | 22 | 45 | 32 | 20 |
| R-46 | Secondary | Karvounari | 79 | 45 | 42 | 32 |
| R-47 | Secondary | Karvounari | 103 | 55 | 45 | 40 |
| R-48 | Secondary | Karvounari | 286 | 70 | 35 | 45 |
| R-49 | Secondary | Karvounari | 229 | 75 | 47 | 25 |
| R-50 | Secondary | Karvounari | 147 | 45 | 45 | 28 |
| R-51 | Secondary | Karvounari | 128 | 50 | 45 | 22 |
| R-52 | Secondary | Karvounari | 312 | 75 | 50 | 32 |
| R-53 | Secondary | Karvounari | 103 | 75 | 40 | 20 |
| R-54 | Secondary | Karvounari | 201 | 62 | 50 | 25 |
| R-55 | Secondary | Karvounari | 200 | 55 | 50 | 28 |
| R-60 | Secondary | Kyparissia | 434 | 87 | 60 | 32 |
| R-61 | Secondary | Kyparissia | 64 | 48 | 37 | 20 |
| R-62 | Secondary | Kyparissia | 146 | 49 | 46 | 25 |
| R-63 | Secondary | Kyparissia | 175 | 56 | 43 | 28 |
| R-64 | Secondary | Kyparissia | 130 | 37 | 31 | 19 |
| R-65 | Secondary | Kyparissia | 140 | 68 | 36 | 22 |
| R-66 | Secondary | Kyparissia | 174 | 73 | 41 | 30 |
| R-67 | Secondary | Kyparissia | 124 | 70 | 45 | 14 |
| R-68 | Secondary | Kyparissia | 110 | 66 | 39 | 22 |
| R-69 | Secondary | Kyparissia | 420 | 82 | 68 | 29 |
| R-70 | Secondary | Kyparissia | 262 | 73 | 44 | 35 |
| R-71 | Secondary | Kyparissia | 502 | 79 | 52 | 47 |
| R-72 | Secondary | Kyparissia | 170 | 100 | 35 | 30 |
| R-73 | Secondary | Karytaina | 40 | 50 | 30 | 20 |
| R-74 | Secondary | Kyparissia | 225 | 78 | 56 | 30 |
| R-75 | Secondary | Karvounari | 378 | 97 | 65 | 39 |
| R-76 | Secondary | Agios Ioannis | 169 | 69 | 66 | 30 |
| R-77 | Secondary | Routsi | 240 | 115 | 44 | 25 |
| R-78 | Secondary | Karytaina | 120 | 72 | 55 | 22 |
| R-79 | Secondary | Kyparissia | 43 | 51 | 21 | 19 |
| R-80 | Secondary | Karvounari | 55 | 40 | 38 | 24 |
| R-81 | Secondary | Agios Ioannis | 302 | 78 | 62 | 43 |
| R-82 | Secondary | Routsi | 407 | 81 | 65 | 48 |
| R-83 | Secondary | Karytaina | 160 | 62 | 47 | 36 |
| R-84 | Secondary | Kyparissia | 99 | 50 | 40 | 30 |
| R-85 | Secondary | Karvounari | 288 | 72 | 58 | 43 |
| R-86 | Secondary | Agios Ioannis | 104 | 51 | 41 | 31 |
| R-87 | Secondary | Routsi | 164 | 60 | 50 | 37 |
| R-88 | Secondary | Karytaina | 73 | 46 | 36 | 27 |
| R-89 | Secondary | Kyparissia | 82 | 47 | 38 | 28 |
| F-90 | Secondary | Karvounari | 38 | 37 | 29 | 22 |
| R-91 | Secondary | Agios Ioannis | 98 | 50 | 40 | 30 |
| R-92 | Secondary | Routsi | 200 | 64 | 51 | 38 |
| R-93 | Secondary | Karytaina | 90 | 49 | 39 | 29 |
| R-94 | Secondary | Kyparissia | 262 | 70 | 56 | 42 |
| R-1X | Secondary | Karvounari | 120 | 54 | 43 | 32 |
| R-74b | Secondary | Agios Ioannis | 1635 | 129 | 103 | 77 |

**Table B. Details of the hammerstones utilised in the experiment.**

| **Hammerstone** | **Mass (g)** | **Length (mm)** | **Width (mm)** | **Thickness (mm)** |
| --- | --- | --- | --- | --- |
| **H1 (Quartzite)** | 514 | 99 | 70 | 47 |
| **H2 (Radiolarite)** | 257 | 87 | 62 | 41 |
| **H3 (Limestone)** | 279 | 91 | 64 | 43 |
| **H4 (Metamorphic-Quartz)** | 95 | 58 | 43 | 35 |
| **H5 (Quartzite)** | 623 | 108 | 76 | 51 |
| **H6 (Flint)** | 476 | 106 | 75 | 50 |
| **H7 (Limestone)** | 734 | 120 | 84 | 56 |
| **H8 (Flint)** | 123 | 72 | 51 | 34 |

**Table C. Experimental knapping sequences.** Table A. Experimental knapping sequences. This table lists all cores and their characteristics, including the assigned knapper (T = knapper with theoretical knowledge, E = expert knapper), the applied technique (B = bipolar, FH = freehand, B.A.A = bipolar anvil-assisted), and relevant volumetric and technological attributes. The number of trials (persistence) does not necessarily exceed the number of resulting flakes, as multiple flakes can detach from a single blow. Note also that the trial count refers only to unsuccessful strikes preceding flake production; successful blows are not recorded. Consequently, in some cases, flakes were produced with no recorded failed attempts, and some cores show more flakes than trials.

| **Technique** | **Ref. core** | **Volume core mm^3^** | **Mass (g)** | **Flakes FH** | **Flakes**  **B** | **Knapper** | **Persistence (N of trials)** | **Reduction sequence** |
| --- | --- | --- | --- | --- | --- | --- | --- | --- |
| **B+FH** | R17 | 48.75 | 60 | 2 | 2 | T | B 5 FH 2 | B: Vertical Axial - Horizontal axial FH: Unifacial Unipolar |
| **B+FH** | R19 | 45 | 82 | 0 | 0 | T | B 1 FH 3 | B: Unipolar Peripheral/Semi peripheral FH: Bifacial Unipolar |
| **B+FH** | R72 | 105 | 170 | 26 | 6 | E | B 2 FH 14 | B: Unifacial Bipolar FH: Bifacial Unipolar |
| **B+FH+B+FH** | R1X | 115.2 | 190 | 10 | 1 | T | B 10 FH 0 | B: Unifacial UnipolarFH: Bifacial Unipolar |
| **FH+B** | R22 | 165 | 196 | 5 | 1 | T | FH 19 B 6 | B: Unifacial Unipolar FH: Unifacial Unipolar |
| **FH+B** | R30 | 146.25 | 148 | 3 | 1 | T | FH 9 B 15 | FH: Unifacial Unipolar B: Bifacial Orthogonal |
| **FH+B** | R53 | 60 | 103 | 6 | 0 | T | FH 19 B 1 | FH: Bifacial Unipolar B: Unifacial Unipolar |
| **FH+B.A.A.** | R68 | 56.628 | 110 | 11 | 3 | T | FH 42 B.A.A 12 | FH: Unifacial Unipolar B.A.A.: Unifacial Unipolar |
| **FH+B+FH** | R66 | 89.79 | 174 | 4 | 0 | T | FH 7 B 4 | FH Unifacial Alternate B Horizontal axial |
| **Total flakes** |  |  |  | 67 | 11+3 |  |  |  |
| **B** | R3 | 346.5 | 468 |  | 0 | T | 11 | Vertical Axial |
| **B** | R11 | 80 | 161 |  | 0 | T | 10 | Vertical Axial |
| **B** | R24 | 8.976 | 34 |  | 2 | T | 2 | Horizontal axial |
| **B** | R37 | 81.4 | 63 |  | 3 | T | 22 | Horizontal axial |
| **B** | R45 | 28.8 | 22 |  | 6 | T | 20 | Horizontal axial |
| **B** | R46 | 60.48 | 79 |  | 4 | T | 30 | Horizontal axial |
| **B** | R64 | 21.793 | 130 |  | 9 | T | 23 | Horizontal axial |
| **B** | R79 | 20.349 | 43 |  | 3 | E |  | Horizontal axial |
| **B** | F90 | 26.784 | 38 |  | 1 | E |  | Vertical Axial |
| **Total flakes** |  |  |  |  | 28 |  |  |  |
| **B.A.A.** | R73 | 30 | 40 |  | 15 | T | 87 | Oblique |
| **FH** | R1 | 1165.5 | 1892 | 1 |  | E | 4 | Unidirectional |
| **FH** | R2 | 253.5 | 274 | 9 |  | T | 16 | Narrow face alternate |
| **FH** | R5 | 302.4 | 357 | 12 |  | E | 8 | Unidirectional-Centripetal |
| **FH** | R13 | 75 | 190 | 5 |  | T | 5 | Unidirectional |
| **FH** | R15 | 70 | 196 | 0 |  | E | 1 | NA |
| **FH** | R28 | 54.45 | 77 | 0 |  | E | 4 | NA |
| **FH** | R31 | 112.8 | 125 | 7 |  | T | 18 | Unidirectional |
| **FH** | R32 | 255 | 321 | 4 |  | T | 8 | Unifacial Alternate |
| **FH** | R33 | 242.775 | 277 | 0 |  | E | 1 | Unidirectional |
| **FH** | R34 | 193.8 | 350 | 1 |  | T | 5 | Unidirectional |
| **FH** | R39 | 92.95 | 112 | 6 |  | E | 2 | Unidirectional |
| **FH** | R44 | 297.6 | 443 | 1 |  | E | 3 | Unidirectional |
| **FH** | R47 | 99 | 103 | 11 |  | T | 25 | Unifacial Alternate |
| **FH** | R52 | 120 | 107 | 14 |  | E | 17 | Unidirectional |
| **FH** | R61 | 35.52 | 64 | 0 |  | T |  | Unidirectional |
| **FH** | R63 | 67.424 | 95 | 10 |  | E | 21 | Unifacial Alternate |
| **FH** | R67 | 44.1 | 124 | 1 |  | E | 6 | Unidirectional |
| **FH** | R70 | 112.42 | 262 | 3 |  | T | 5 | Unifacial Bidirectional - Alternate |
| **FH** | R74b | 950.4 | 1635 | 14 |  | E | 14 | Unidirectional |
| **FH** | R77 | 126.5 | 240 | 0 |  | E |  | Unidirectional |
| **FH** | R80 | 36.48 | 55 | 0 |  | E |  | Unidirectional |
| **FH** | R82 | 291.2 | 407 | 1 |  | E |  | Unidirectional |
| **FH** | R83 | 90 | 160 | 4 |  | E | 4 | Unidirectional |
| **FH** | R84 | 49.4 | 99 | 7 |  | E | 5 | Unidirectional |
| **FH** | R85 | 168 | 288 | 2 |  | E | 2 | Unidirectional |
| **FH** | R86 | 73.5 | 104 | 5 |  | E | 4 | Unidirectional |
| **FH** | R87 | 84.816 | 164 | 28 |  | E | 15 | Unidirectional |
| **FH** | R88 | 42 | 73 | 8 |  | E | 8 | Unidirectional |
| **FH** | R89 | 52.8 | 82 | 6 |  | E | 4 | Unidirectional |
| **FH** | R91 | 49.4 | 98 | 6 |  | E | 5 | Unidirectional |
| **FH** | R93 | 42.9 | 90 | 21 |  | E |  | Centripetal |
| **FH** | R94 | 155.25 | 262 | 10 |  | E |  | Centripetal |
| **Total flakes** |  |  |  | 197 |  |  |  |  |

**Table D. List of refitted experimental cores.**

The list includes the name of the core, maximum dimensions and number of flakes produced with a length of 15mm or more.

| **Name** | **L (mm)** | **W (mm)** | **T (mm)** | **N Flakes** |
| --- | --- | --- | --- | --- |
| **R-22** | 75 | 55 | 40 | 5 |
| **R-31** | 60 | 47 | 40 | 7 |
| **R-39** | 65 | 65 | 22 | 6 |
| **R-52** | 75 | 50 | 32 | 14 |
| **R-63** | 56 | 43 | 28 | 10 |
| **R-67** | 70 | 45 | 14 | 1 |
| **R-68** | 66 | 39 | 22 | 11 |
| **R-70** | 73 | 44 | 35 | 3 |
| **R-72** | 100 | 35 | 30 | 26 |
| **R-79** | 51 | 21 | 19 | 3 |
| **R-87** | 76 | 62 | 18 | 28 |
| **R-91** | 65 | 38 | 20 | 6 |
| **R-93** | 65 | 33 | 20 | 21 |

**
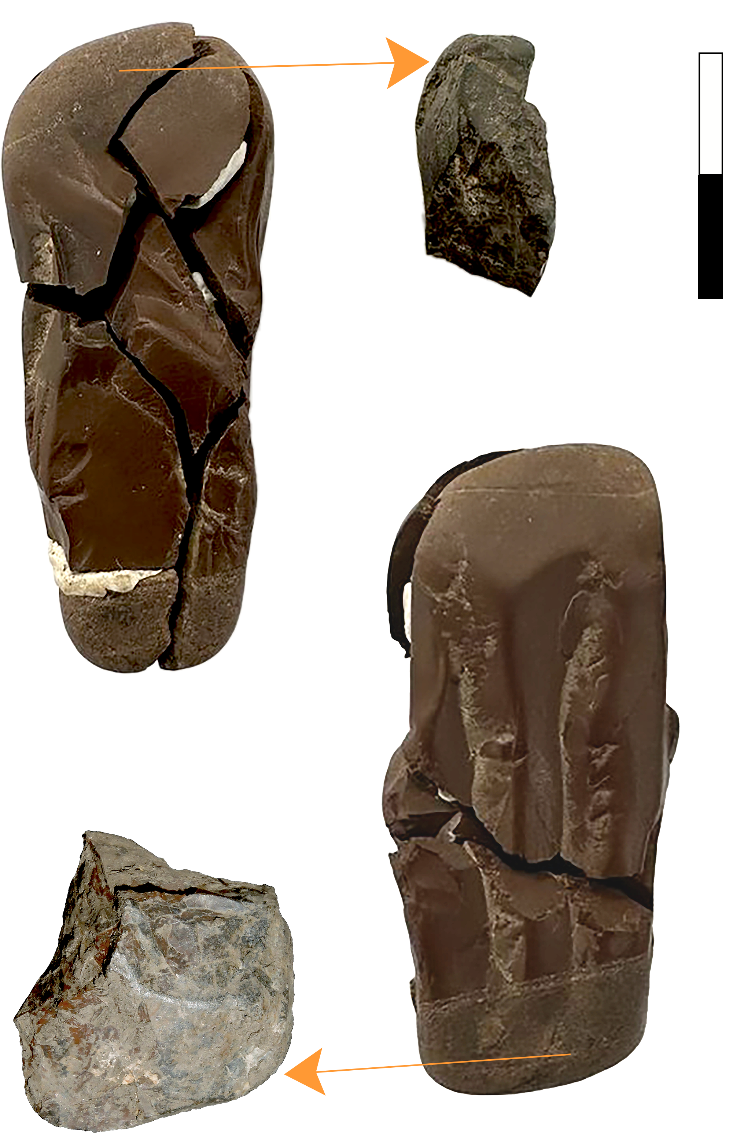
**

**Fig. A. experimental refitted core exploited through bipolar technique and comparisons with two flakes from the archaeological assemblage.**


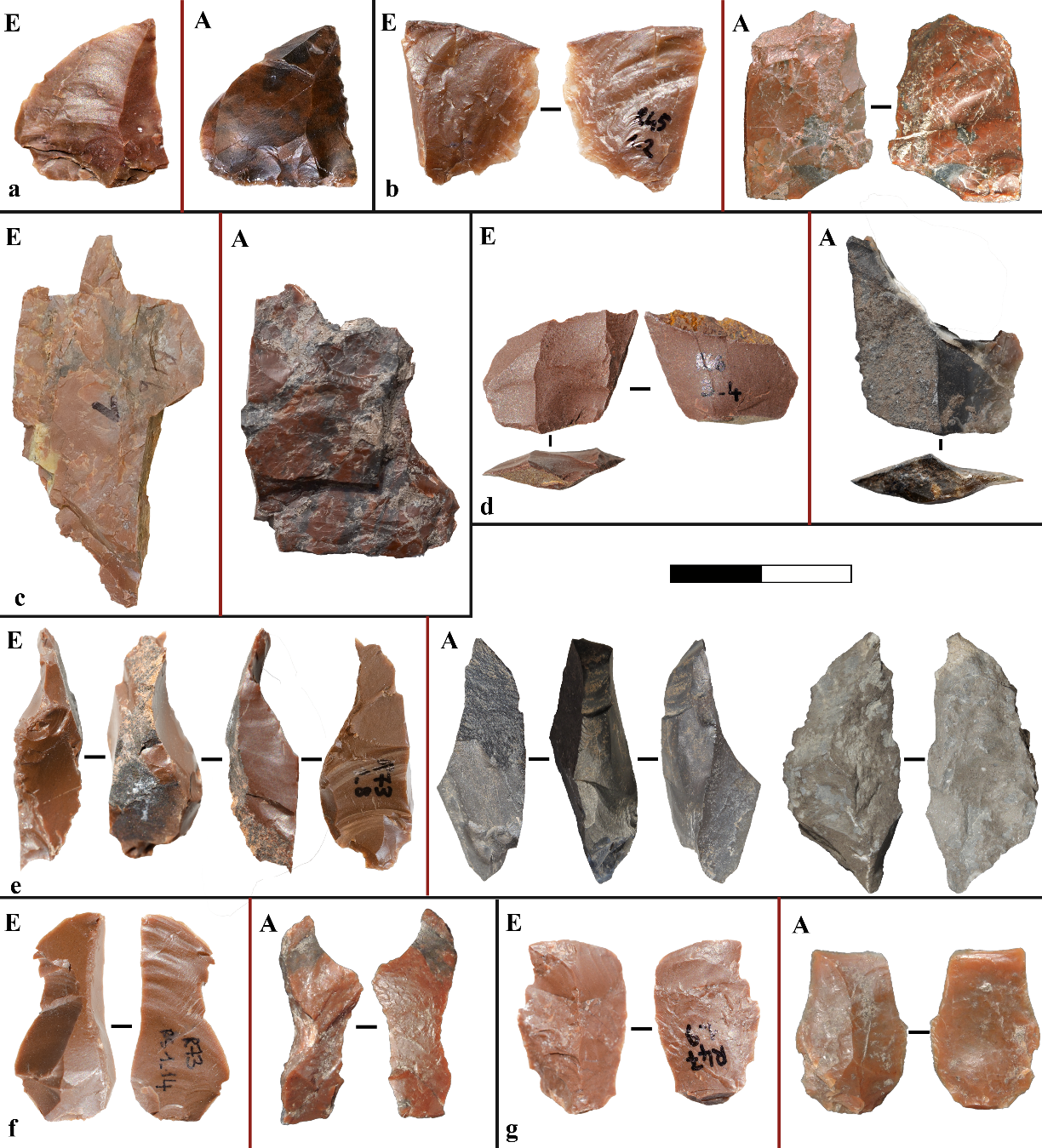


**Fig. B. Experimental (E) and archaeological (A) flakes**

a) Freehand small triangular flakes obtained through centripetal reduction. b) Bipolar flake with pronounced and irregular ripples and a flat ventral surface. c) Flat pieces without typical flake features produced through bipolar percussion demonstrate this technique's varied results. d) Proximal fragment of a freehand regular elongated flake broken for an internal dishomogeneity (E= cleavage, A= calcite vein). e) An elongated piece with a subrectangular section and pointed extremities, characterised by irregular ripples resulting from bipolar reduction. f) Bipolar bladelets. g) A flake produced by freehand knapping, featuring a distinct central ridge, reflecting the technique’s suitability to create well-defined edges and shapes.


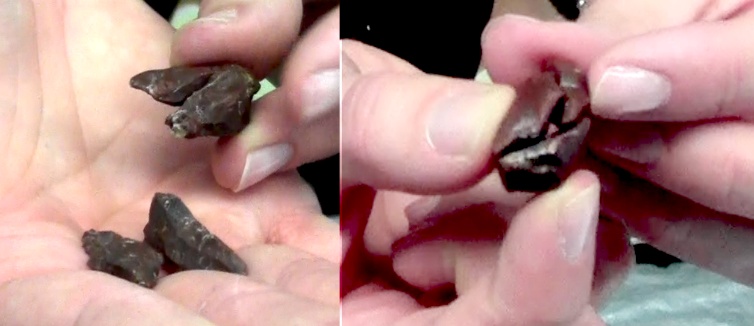


**Fig. C. Fully reduced core.**

The final strike broke the core into four elongated pieces.


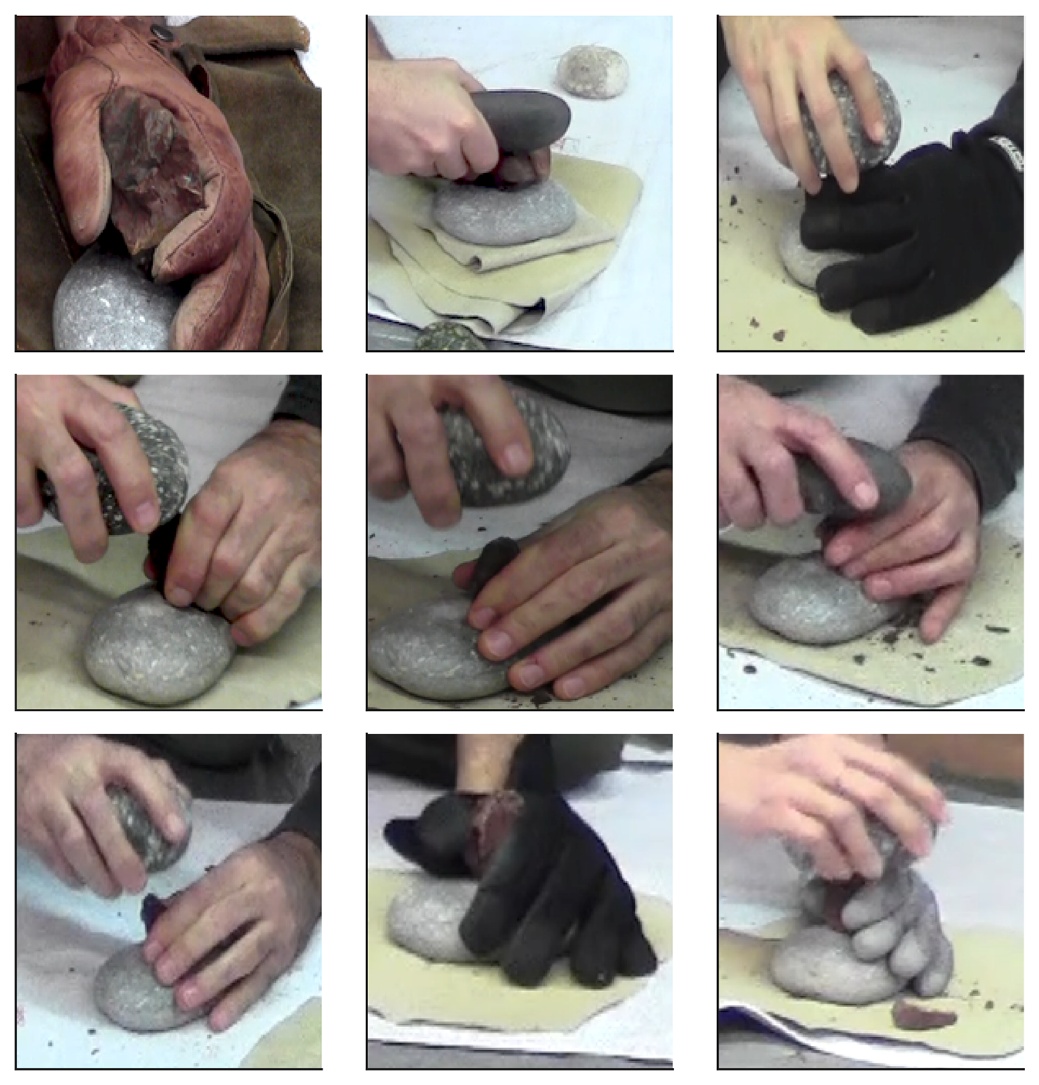


**Fig. D. Position of the three elements (hammerstone, core, and anvil) during bipolar-on-anvil percussion.**

The collage illustrates nine cores reduced using the bipolar technique. During the overall experiment, hammerstones were typically positioned centrally to optimise compression, except for two instances in which an a slight eccentric position was used. These eccentric positions resulted in no noticeable differences in flake morphology.


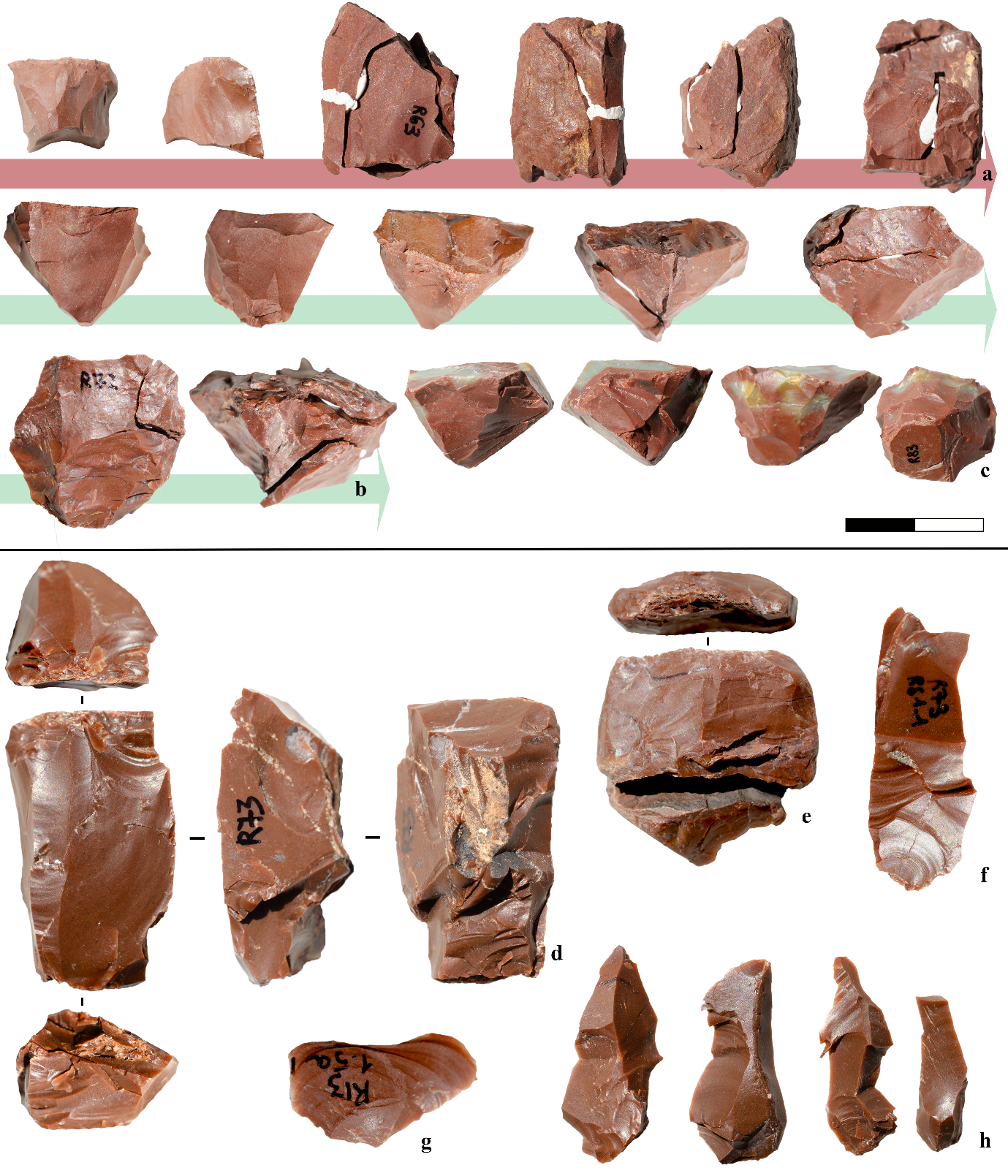


**Fig. E. Freehand cores and bipolar-on-anvil artefacts.**

a-b) Small freehand cores with flake refits. c) Exhausted freehand core, comparable to Fig. 11 No. 1 in the main article. d) Bipolar core showing crushing on edges adjacent to percussive elements (hammerstone and anvil). e) Bipolar core fractured along a cleavage plane, with percussion marks on the edge adjacent to the hammerstone. f) Bipolar flake with bidirectional ripples on the ventral surface. g) Hinge bulb of percussion. h) Elongated flakes with proportionally narrow platforms typical of bipolar reduction, as the result of the PCA identified in PC2 values.

# Statistics

## Categorical PCA (without ripples).

| **Table E. CATPCA Component Loadings.** | | | | | | |  |  |
| --- | --- | --- | --- | --- | --- | --- | --- | --- |
|  | | Dimension | | | | |  |  |
|  |  | 1 | | 2 | 3 | |  |  |
| Bulb | | -.502 | | .345 | -.659 | |  |  |
| Flake_Termination | | .639 | | .628 | -.130 | |  |  |
| Sharp_Edges | | -.821 | | -.319 | .035 | |  |  |
| Central | | -.344 | | .493 | .708 | |  |  |
| Platform_Preparation | | -.526 | | .608 | -.047 | |  |  |
| **Table F. CATPCA Model Summary.** | | | | | | |  |  |
| Dimension | | Cronbach's Alpha | | Variance Accounted For | | | | |
|  |  |  |  | Total (Eigenvalue) | | | % of Variance | |
| 1 | | .528 | | 1.731 | | | 34.628 | |
| 2 | | .232 | | 1.228 | | | 24.555 | |
| 3 | | -.058 | | .955 | | | 19.108 | |
| Total | | .931^a^ | | 3.915 | | | 78.291 | |
|  | | | | | | | | |

**Fig. F. CATPCA excluding the variable “ripples”.**

## PCA and CATPCA with Control Group (B.A.A.)

The results of the Mann-Whitney U tests indicate that there are no statistically significant differences between Bipolar knapping (Group 1) and Bipolar anvil-assisted knapping (Group 4) in terms of the examined Principal Components (PC1, PC2) and Categorical Principal Components (CATPC1, CATPC2). While minor variations exist, as reflected in the range of Z-scores (0.267 to 1.021), none of the differences reach statistical significance (p > 0.05). This suggests that both knapping techniques did not lead to distinct patterns in the dataset. Overall, these findings imply that while bipolar and anvil-assisted bipolar knapping may involve different mechanical applications, their resultant flakes share similar technological attributes, indicating a degree of interchangeability or overlap in their reduction strategies, at least under the conditions of our experiment that involved the radiolarite from the Megalopolis basin.

**Table G. Mann-Whitney U Test results comparing PCs and CATPCs between (Bipolar) and Bipolar Anvil-Assisted.** All p-values (p > 0.05) indicate no statistically significant differences between the two groups, suggesting that both knapping techniques produce flakes with similar attributes.

|  | PC1 | PC2 | CATPC1 | CATPC2 |  |
| --- | --- | --- | --- | --- | --- |
| Mann-Whitney U | 159 | 124 | 120 | 103 |  |
| Wilcoxon W | 170 | 135 | 131 | 114 |  |
| Z | 0.941636 | 0.266501 | 0.411907 | 1.020813 |  |
| Asymp. Sig. | 0.346379 | 0.789853 | 0.680407 | 0.307343 |  |

| **Table H. PCA with control group. Total Variance Explained.** | | | | | | |
| --- | --- | --- | --- | --- | --- | --- |
| Component | Initial Eigenvalues | | | Extraction Sums of Squared Loadings | | |
|  | Total | % of Variance | Cumulative % | Total | % of Variance | Cumulative % |
| 1 | 3.155 | 63.101 | 63.101 | 3.155 | 63.101 | 63.101 |
| 2 | .800 | 15.997 | 79.098 | .800 | 15.997 | 79.098 |
| 3 | .492 | 9.841 | 88.940 | .492 | 9.841 | 88.940 |
| 4 | .335 | 6.692 | 95.631 | .335 | 6.692 | 95.631 |
| 5 | .218 | 4.369 | 100.000 |  |  |  |
|  | | | | | | |
| \| **Table I. PCA Component Matrix.** \| \| \| \| \| \| --- \| --- \| --- \| --- \| --- \| \|  \| Component \| \| \| \| \| 1 \| 2 \| 3 \| 4 \| \| Length \| .723 \| .583 \| .124 \| .336 \| \| Width \| .818 \| -.190 \| .451 \| -.242 \| \| Thickness \| .854 \| .315 \| -.099 \| -.270 \| \| P_Depth \| .816 \| -.149 \| -.510 \| -.046 \| \| P_Width \| .754 \| -.550 \| .057 \| .297 \| \| Extraction Method: Principal Component Analysis. \| \| \| \| \| \|  \| \| \| \| \| | | | | | | |

##
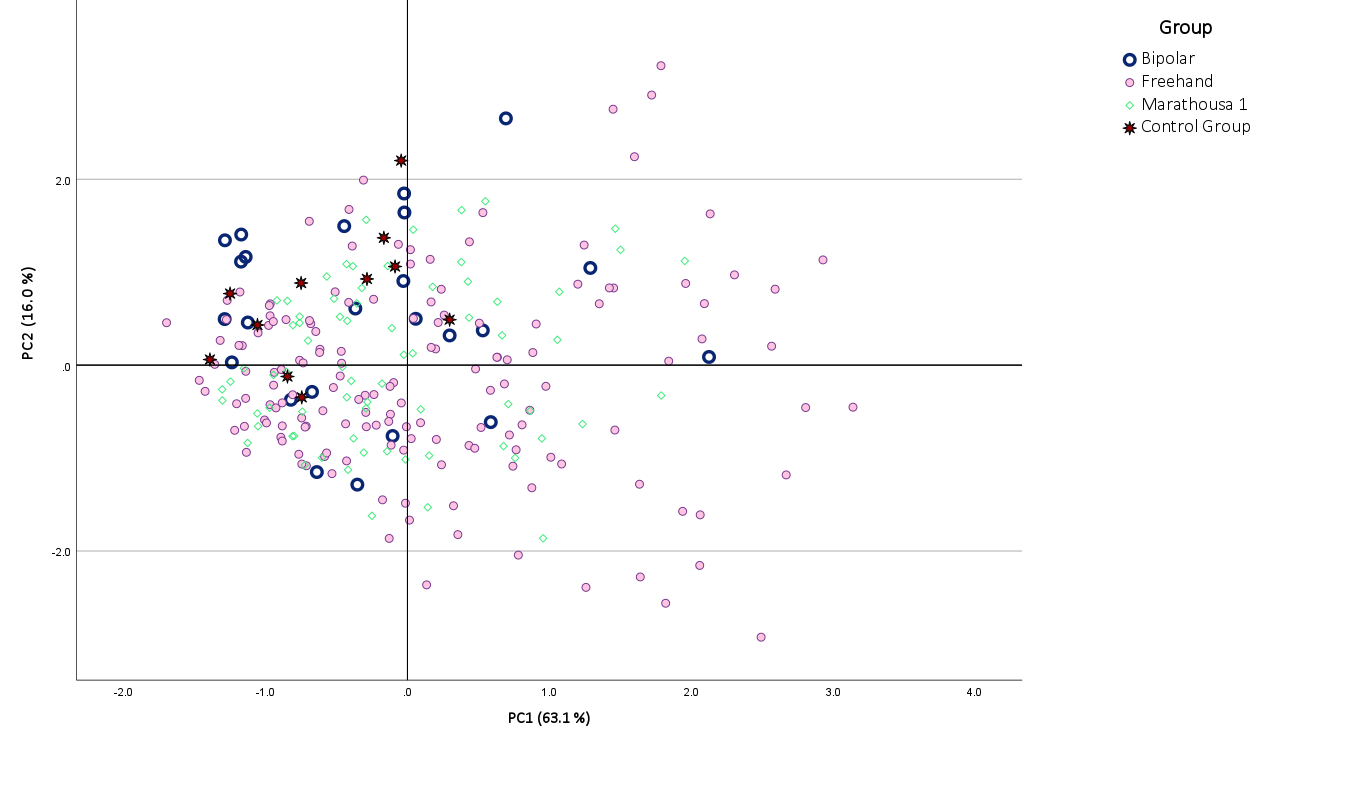


**Fig. G. PCA with the control group (B.A.A.) overlapping in Bipolar variance.**

| \| **Table J.CATPCA model summary.** \| \| \| \| \| --- \| --- \| --- \| --- \| \| Dimension \| Cronbach's Alpha \| Variance Accounted For \| \| \| Total (Eigenvalue) \| % of Variance \| \| 1 \| .570 \| 1.904 \| 31.740 \| \| 2 \| .335 \| 1.387 \| 23.114 \| \| 3 \| .020 \| 1.017 \| 16.954 \| \| 4 \| -.484 \| .712 \| 11.872 \| \| Total \| .961^a^ \| 5.021 \| 83.680 \| \|  \| \| \| \| |
| --- | --- | --- | --- | --- | --- | --- | --- | --- | --- | --- | --- | --- | --- | --- | --- | --- | --- | --- | --- | --- | --- | --- | --- | --- | --- | --- | --- | --- | --- | --- | --- | --- | --- | --- |

| **Table K. Correlations Transformed Variables.** | | | | | | |
| --- | --- | --- | --- | --- | --- | --- |
|  | Bulb | Flake_Termination | Sharp_Edges | Central | Platform_Preparation | Ripples |
| Bulb | 1.000 | -.047 | .216 | .039 | .264 | .440 |
| Flake_Termination | -.047 | 1.000 | -.531 | -.025 | -.009 | .017 |
| Sharp_Edges | .216 | -.531 | 1.000 | .075 | .202 | .116 |
| Central | .039 | -.025 | .075 | 1.000 | .190 | .077 |
| Platform_Preparation | .264 | -.009 | .202 | .190 | 1.000 | .302 |
| Ripples | .440 | .017 | .116 | .077 | .302 | 1.000 |
| Dimension | 1 | 2 | 3 | 4 | 5 | 6 |
| Eigenvalue | 1.904 | 1.387 | 1.017 | .712 | .554 | .425 |


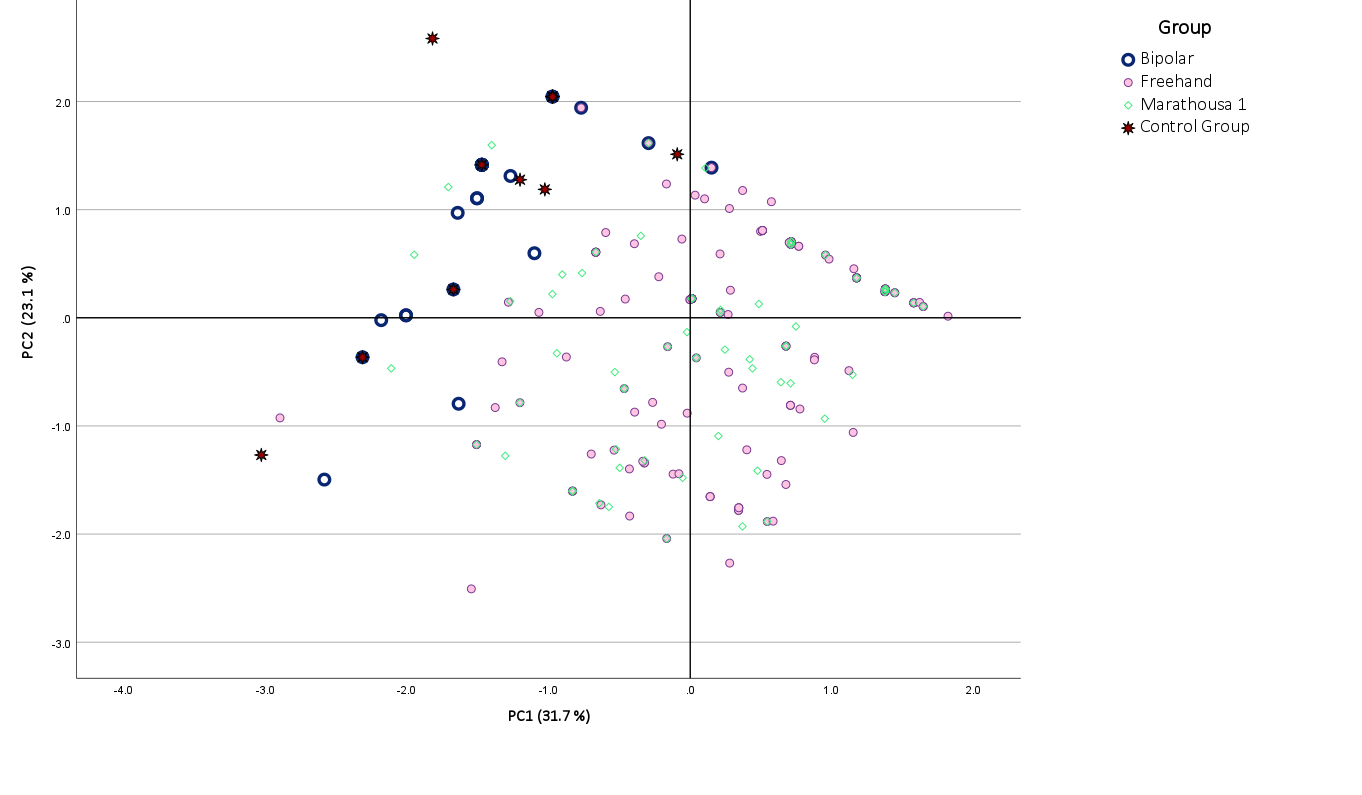


**Fig. H. CATPCA with Control Group (B.A.A) overlapping with Bipolar.**

The extreme in PC1-/PC2+ indicates a B.A.A. flake with three sharp edges, feathered termination, hinged bulb, absence of central convexity, and absence of ripples.

## Tests

**Kruskal-Wallis Test**

| **Table L. Ranks considering the three groups (1. Bipolar, 2. Freehand, 3. Marathousa 1)** | | | |
| --- | --- | --- | --- |
|  | Group | N | Mean Rank |
| PC1 | 1 | 35 | 104.60 |
|  | 2 | 155 | 138.58 |
|  | 3 | 71 | 127.46 |
|  | Total | 261 |  |
| PC2 | 1 | 35 | 176.86 |
|  | 2 | 155 | 119.57 |
|  | 3 | 71 | 133.34 |
|  | Total | 261 |  |
| CATPC1 | 1 | 35 | 33.87 |
|  | 2 | 155 | 151.78 |
|  | 3 | 71 | 133.51 |
|  | Total | 261 |  |
| CATPC2 | 1 | 35 | 197.56 |
|  | 2 | 155 | 124.51 |
|  | 3 | 71 | 112.35 |
|  | Total | 261 |  |

**Table M. Results of the Kruskal-Wallis Test, including the H values, the degree of freedom (df) and the P values (Asymp. Sig.).**

|  | | PC1 | | PC2 | | CATPC1 | | CATPC2 | |
| --- | --- | --- | --- | --- | --- | --- | --- | --- | --- |
| Kruskal-Wallis H | | 6.000 | | 16.535 | | 69.821 | | 32.709 | |
| df | | 2 | | 2 | | 2 | | 2 | |
| Asymp. Sig. | | .050 | | <.001 | | <.001 | | <.001 | |
| **Mann-Whitney Test**  **Table N. Ranks considering two groups (1. Bipolar, 2 Freehand).** | | | | | | | | |  |
|  | Group | | N | | Mean Rank | | Sum of Ranks | |  |
| PC2 | 1 | | 35 | | 129.14 | | 4520.00 | |  |
|  | 2 | | 155 | | 87.90 | | 13625.00 | |  |
|  | Total | | 190 | |  | |  | |  |
| CATPC1 | 1 | | 35 | | 26.54 | | 929.00 | |  |
|  | 2 | | 155 | | 111.07 | | 17216.00 | |  |
|  | Total | | 190 | |  | |  | |  |
| CATPC2 | 1 | | 35 | | 140.34 | | 4912.00 | |  |
|  | 2 | | 155 | | 85.37 | | 13233.00 | |  |
|  | Total | | 190 | |  | |  | |  |

**Table O. Results of the Mann-Whitney test, including the U values, the W value, Z score and the P values (Asymp. Sig.).**

|  | PC2 | CATPC1 | CATPC2 |
| --- | --- | --- | --- |
| Mann-Whitney U | 1535.000 | 299.000 | 1143.000 |
| Wilcoxon W | 13625.000 | 929.000 | 13233.000 |
| Z | -4.007 | -8.217 | -5.344 |
| Asymp. Sig. (2-tailed) | <.001 | <.001 | <.001 |

**Mann-Whitney Test**

| **Table P. Ranks considering two groups (1. Bipolar, 3. Marathousa 1)** | | | | |
| --- | --- | --- | --- | --- |
|  | Group | N | Mean Rank | Sum of Ranks |
| PC2 | 1 | 35 | 65.71 | 2300.00 |
|  | 3 | 71 | 47.48 | 3371.00 |
|  | Total | 106 |  |  |
| CATPC1 | 1 | 35 | 25.33 | 886.50 |
|  | 3 | 71 | 67.39 | 4784.50 |
|  | Total | 106 |  |  |
| CATPC2 | 1 | 35 | 75.21 | 2632.50 |
|  | 3 | 71 | 42.80 | 3038.50 |
|  | Total | 106 |  |  |

**Table Q.** **Results of the Mann-Whitney test, including the U values, the W value, the Z score and the P values (Asymp. Sig.).**

|  | PC2 | CATPC1 | CATPC2 |
| --- | --- | --- | --- |
| Mann-Whitney U | 815.000 | 256.500 | 482.500 |
| Wilcoxon W | 3371.000 | 886.500 | 3038.500 |
| Z | -2.872 | -6.627 | -5.108 |
| Asymp. Sig. (2-tailed) | .004 | <.001 | <.001 |

**Mann-Whitney Test**

**Table R. Ranks considering two groups (2 Freehand, 3 Marathousa 1).**

|  | Group | N | Mean Rank | Sum of Ranks |
| --- | --- | --- | --- | --- |
| PC2 | 2 | 155 | 109.67 | 16999.00 |
|  | 3 | 71 | 121.86 | 8652.00 |
|  | Total | 226 |  |  |
| CATPC1 | 2 | 155 | 118.71 | 18400.50 |
|  | 3 | 71 | 102.12 | 7250.50 |
|  | Total | 226 |  |  |
| CATPC2 | 2 | 155 | 117.14 | 18156.50 |
|  | 3 | 71 | 105.56 | 7494.50 |
|  | Total | 226 |  |  |

**Table S.** **Results of the Mann-Whitney test, including the U values, the W value, the Z score and the P values (Asymp. Sig.).**

|  | PC2 | CATPC1 | CATPC2 |
| --- | --- | --- | --- |
| Mann-Whitney U | 4909.000 | 4694.500 | 4938.500 |
| Wilcoxon W | 16999.000 | 7250.500 | 7494.500 |
| Z | -1.301 | -1.772 | -1.237 |
| Asymp. Sig. (2-tailed) | .193 | .076 | .216 |
